# Discovering the opposite shore: How did hominins cross sea straits?

**DOI:** 10.1371/journal.pone.0252885

**Published:** 2021-06-30

**Authors:** Ericson Hölzchen, Christine Hertler, Ana Mateos, Jesús Rodríguez, Jan Ole Berndt, Ingo J. Timm

**Affiliations:** 1 Faculty of Biosciences, Department of Paleobiology and Environment, Institute of Ecology, Evolution and Diversity, Goethe University Frankfurt, Frankfurt am Main, Germany; 2 The Role of Culture in Early Expansion of Humans (ROCEEH), Senckenberg Research Institute, Frankfurt am Main, Germany; 3 The Role of Culture in Early Expansion of Humans (ROCEEH), Heidelberg Academy of Sciences, Heidelberg, Germany; 4 National Research Center on Human Evolution (CENIEH), Burgos, Spain; 5 Chair for Business Informatics I Trier Lab for Social Simulation (TRILABS), Trier University, Trier, Germany; 6 German Research Center for Artificial Intelligence (DFKI) Cognitive Social Simulation (Branch Trier), Trier University, Trier, Germany; Max Planck Institute for the Science of Human History, GERMANY

## Abstract

Understanding hominin expansions requires the comprehension of movement processes at different scales. In many models of hominin expansion these processes are viewed as being determined by large-scale effects, such as changes in climate and vegetation spanning continents and thousands or even millions of years. However, these large-scale patterns of expansions also need to be considered as possibly resulting from the accumulation of small-scale decisions of individual hominins. Moving on a continental scale may for instance involve crossing a water barrier. We present a generalized agent-based model for simulating the crossing of a water barrier where the agents represent the hominin individuals. The model can be configured to represent a variety of movement modes across water. Here, we compare four different behavioral scenarios in conjunction with a set of water barrier configurations, in which agents move in water by either paddling, drifting, swimming or rafting. We introduce the *crossing-success-rate* (CSR) to quantify the performance in water crossing. Our study suggests that more focus should be directed towards the exploration of behavioral models for hominins, as directionality may be a more powerful factor for crossing a barrier than environmental opportunities alone. A prerequisite for this is to perceive the opposite shore. Furthermore, to provide a comprehensive understanding of hominin expansions, the CSR allows for the integration of results obtained from small-scale simulations into large-scale models for hominin expansion.

## Introduction

The genus *Homo* first appeared in Africa 2.8 Ma [1] and eventually expanded throughout the world. This expansion process occurred in several phases and involved a number of *Homo* species differing in cognitive and technological capabilities. Many of those expansion phases required the hominins to cross water bodies of varying sizes, from large rivers to sea straits [2–5]. The extent to which hominins were able to cross sea straits is debated [5–10] and agent-based models can hypothetically show how expansions would look like if crossing sea straits are possible. Thus, investigating the factors influencing the success in crossing a water barrier by hominins having no advanced seafaring technology is essential for debating the possible migration routes during the Palaeolithic.

The purpose of this study is to introduce a generalized agent-based model that simulates hominin movement across a hypothetical, simulated landscape divided by a water barrier. We distinguish the ability of hominins to cross water barriers in the form of scenarios. In the model we therefore speak of agents instead of a particular hominin species. These represent levels of physiological and cultural performance, expressed as water movement skills. The model can be configured to represent a variety of water movement skills. Here, we compare four scenarios ranging from paddling to rafting.

Our study presents the first agent-based model for simulating small-scale hominin sea crossing. We explore the technical functionality of the model in a pilot study, which can be transferred to geographically explicit settings in subsequent steps, and test the features of the model as a prerequisite to distinguish the effects evoked by the model design from the results of behavioral alternatives for the agents. The results will then be integrated into large-scale simulations in the future. With the agent-based simulation, we provide an experimental context to the numerous conceptual models for hominin sea crossing from archaeology and paleoanthropology (e.g., [2, 11–14]).

Hominin expansion is generally represented as a large-scale spatio-temporal process being driven by demographic and/or environmental factors and in general, as following a diffusion-like mechanism [15–24].

Models involving low spatial resolution favor terrestrial routes over those involving the crossing of water barriers, sometimes to such an extent that non-terrestrial routes are entirely omitted from further consideration. In addition to the spatial resolution, the temporal resolution determines which dynamic processes are represented in the model. For example, movements across water barriers are reduced to a “yes” or “no” decision which must be decided prior to the modeling procedure [18–20, 23]. Therefore, we need to model water crossings at a sufficiently small spatio-temporal resolution to represent the spatial properties of a water barrier and to address the dynamical properties of movements across a water barrier in an adequate way. For this purpose, we introduce the crossing success rate (CSR) as a means to quantify the crossing success; this is based on the ratio of successful to failed agent accumulations on the opposite shore, where such accumulations are equivalent to a minimal definition of a forming founder population. By using the CSR, we are able to compare different behavioral scenarios and their effects on the crossing success via the basis of small-scale movement decisions. The model introduced here is exclusively intended for simulating the crossing of a water barrier. Other factors are kept to a minimum, for instance, the movement on land has the sole purpose of enabling the agents to enter the water. Behavioral scenarios represent the different means of crossing a water barrier, ranging from basic paddling to directed rafting. The CSR quantifies the success of a given movement mode under specific configuration of the water barrier.

## Model design

In the model, we simulate the movement of agents on land and across a water barrier and quantify this process by utilizing the output variable, CSR. When describing the model parameters, we use the terminology following Montgomery and Douglas [25] where the parameters, which may be changed between simulation experiments, are called “factors”, the factor values are called “levels”, parameters that are fixed are called “constants”, and the output variables are called “responses”.

### Environment in the model

**Fig 1:**
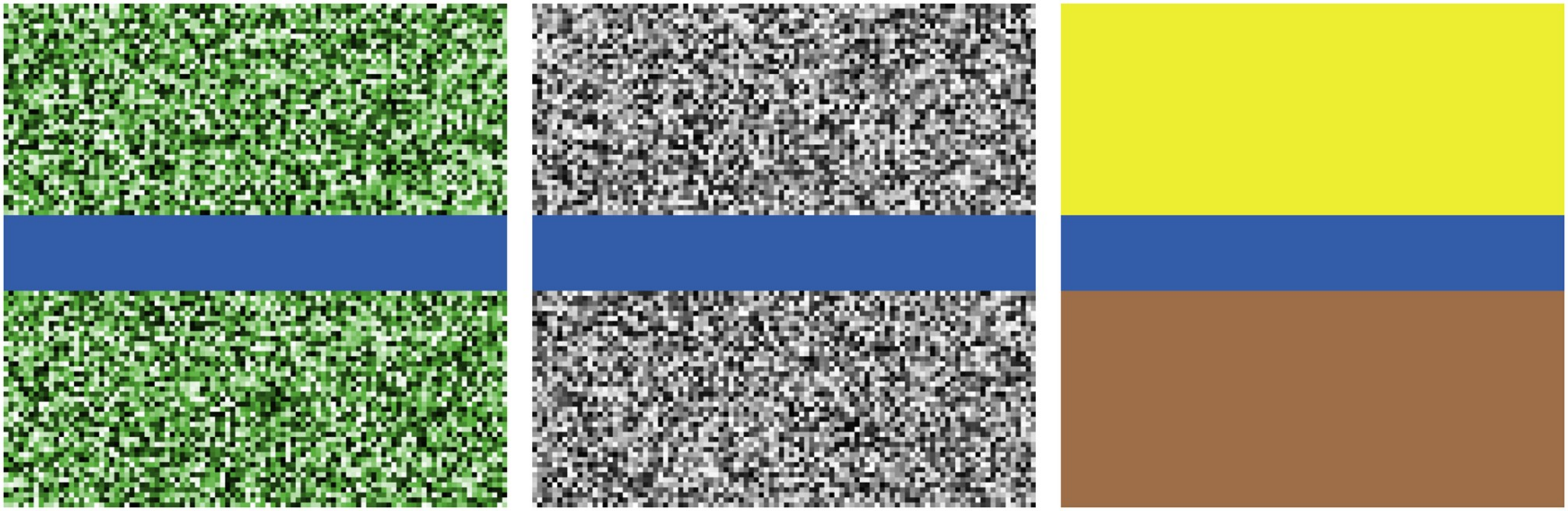
Default map (100 x 100 patches) with random values for resource quality and slope. Resource quality map (left), slope map (middle) and source-target map (right). The source area is shown in brown and the target area in yellow. The water barrier is shown in blue with a size of 15 patches (default level).

The map of the environment in the agent-based model consists of a grid of “patches”. A water barrier deep and wide enough to prevent foot wading separates the source and target areas. Patches are defined by three features: resource quality, slope and water (Fig 1).

Resource qualities refer to the availability of resources for subsistence on land and are represented by a float value of between 0 and 1. A value of 0 means that the patch possesses no resources and a value of 1 means that the patch has resources with maximum quality. Resource qualities of between 0 and 1 represent intermediate amounts. The slope is represented in percentage rise. One patch represents 1 km^2^ of geographical space. Factors for creating the map are given in Table 1. Values for resource qualities and slope are randomly distributed across the land patches on the map.

**Table 1:** Map settings.

Factor	Description	Default level
*water-barrier-size*	defines the size of the water barrier in the random map scenario in kilometers	10 km
*water-temperature-interval*	defines the water temperature in degrees Celsius	24–31°C
*current-speed*	defines the speed of the water current in kilometers per hour	0 km/h (no current)

### Movement behavior and survival of the agents

Hominins, such as *Homo erectus*, were of sufficient body size and proportions [26–29] to have enabled advanced swimming, if they possessed the correct techniques. Moreover, hominins may have crossed water barriers of several kilometers in a manner used by other terrestrial mammal species, for instance, by simple swimming or drifting [30–32], even without advanced techniques or maritime navigation. Upper and middle Palaeolithic hominins may have used rafts to navigate across a water barrier.

**Fig 2:**
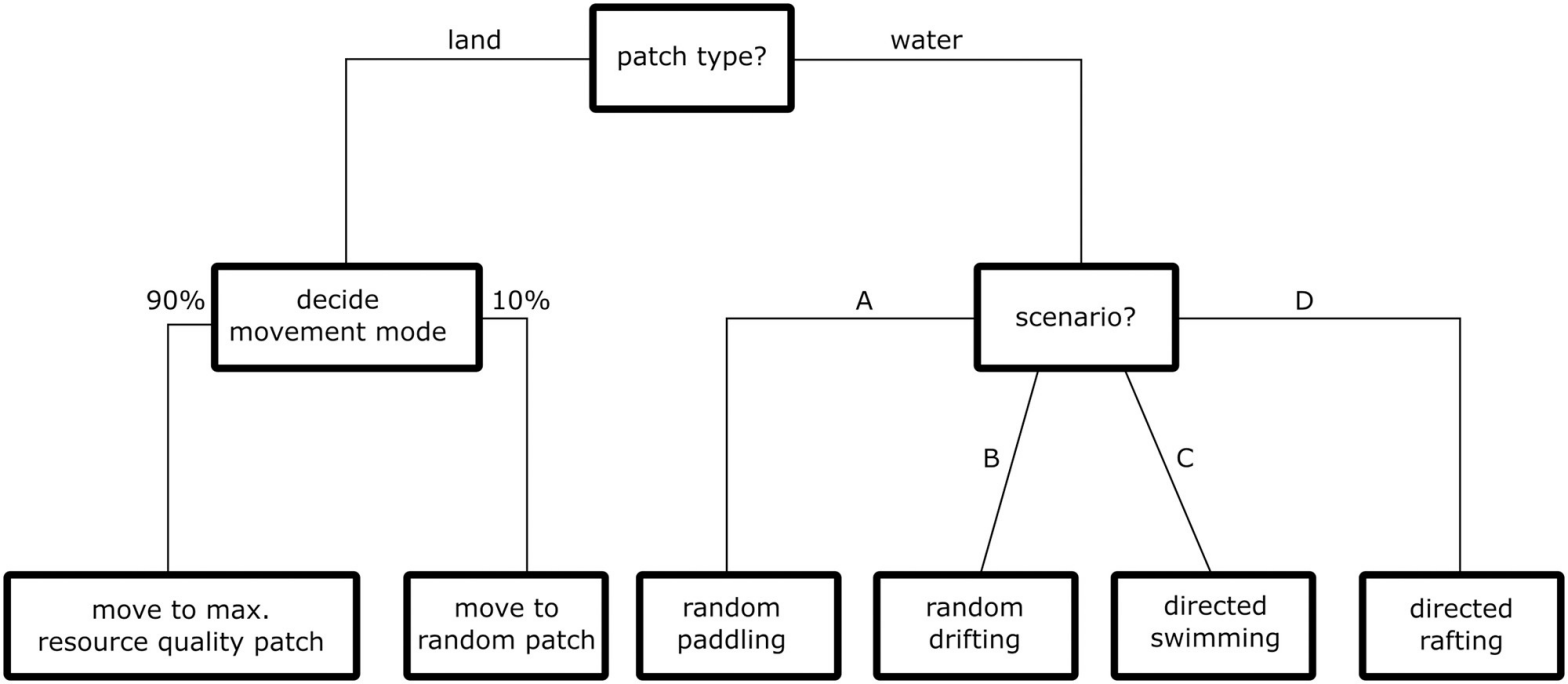
Behavioral decision tree for the scenarios with movement on land (left) and movement in water (right). The movement on land is determined by the resource quality and randomness of the decisions, which is by default set at 10%, whereas the movement in water is determined by the selected scenario.

The agents represent hominin individuals who perform movement on land and in water (Fig 2), whereby “movement” refers to the deplacement that occurs within an hourly scale. This is to be distinguished from deplacements over longer periods of time, e.g., migration or dispersal. As the model focuses solely on movement in the water, we intentionally left out other factors such as complex social interaction, social bonding, collaboration, planning and communication. In addition, the agents are not able to learn or adapt to their environment in this model.

Furthermore, they can leave the map or die. Initially, 1000 individual hominin agents are placed randomly within the source area corresponding to population density estimates for hominins [33].

An agent who is placed on the map selects a target patch to which they direct their movement. The agent perceives neighboring patches within a radius of 20 km. Within this radius, the agent selects a target patch towards which they will head. A higher radius of perception increases the sample of potential target patches surrounding the agent. The radius of perception allows the inclusion of environmental factors such as weather, wave action, clouds, fog and darkness, that affect the perception of the environment into a single value. By default, the agent will select the patch with the highest resource quality with a probability of 90% or a random patch within the perception radius with the remaining 10% probability. Our model, therefore, allows for some degree of decision uncertainty. Due to the random distribution of resource qualities and slope values, the movement on land in the generalized model results in a randomly directed movement with randomized walking speed. We note that the movement would not be random on a map with heterogenous distribution of resource qualities and realistic topography, such as it would be the case in a geographic explicit map. In the generalized model introduced here, the movement on land acts as place holder for more sophisticated movement models (see section “Crossing decision and how to get into the water”).

In order to reach the target patch, the agent moves with a certain movement speed that is determined by the optimal walking speed and the slope value of the local patch. We assume an optimal walking speed of 5 km/h for the agents which closely correspond to the average optimal walking speed of 1.5 m/s of present humans [34]. The effective movement speed equals the optimal movement speed if the slope value is 0. We simplified the effect of the slope by linearly decreasing the effective movement speed with increasing slope value. The minimum speed is 0.05 km/h. This results in effective movement speeds of between 0.05 km/h and 5 km/h on the map. Once the agent has reached their target patch, they will then select a new one and proceed to the following target patch. Agents move into the water, either randomly, due to decision randomness, or when they target a patch on the opposite shore.

On water patches, the agents move by paddling, drifting, swimming or rafting. However, the particular movement mode on the water patches is defined by the specific behavioral scenario described in the section below. In each of the scenarios, the movement on water patches is affected by water current. The water current modulates the performance of paddling, drifting, swimming and rafting by deflecting the direction in which the agents move. The effect is modeled by adding the current speed, with a west to east direction, to the vector of the hominin agent’s movement.

All agents are equipped with an energy storage of 45,000 energy-units. The rate of change in the energetic levels of the agents may differ from empirical caloric (kcal) values during a simulation run. Therefore, we chose the term “energy-unit” to describe the energetic level of the agents. As we focus on the movement in water, we apply a simplified definition for the movement on land. Agents do not spend energy on land; we assume that agents on terrestrial patches receive a sufficient amount of resources and so do not die. This, however, is not the case when on the water patches.

On a water patch, an agent dies of exhaustion when their energy level drops to 0. An agent also loses a basic amount of 62.5 energy-units per hour, corresponding to 1,500 energy-units per day. The equivalent caloric demand is assumed for hominins [35]. A daily reduction by 1,500 energy-units permits the agent to survive for 30 days on water patches. It is worth noting that we assume low capabilities of planning behavior for the agents. The agents, therefore, do not carry food supplies when crossing the water, contrary to the case for a prepared crossing. In addition, as the agents in the water spend additional energy on thermoregulation, we apply the energy loss by thermoregulation as a function of water temperature and exposure time as described by Hayward et al. [36] and modify this to an hourly scale:

$$H_{h}=\left(4.19–0.11T_{w}\right)\cdot 60$$ (1),

where *H*_*h*_ is the energy loss from thermoregulation in energy-units per hour and *T*_*w*_ is the water temperature in degrees Celsius.

In addition, paddling or swimming reduces the energy storage by a rate of 800 energy-units per hour, corresponding to the caloric demand of English Channel swimmers [37], whereas the agent spends its energy solely on thermoregulation when passively drifting. In the case of rafting, the agent spends 383 energy-units per hour on rowing, corresponding to the energy expenditure of competitive rowers [38].

An agent can only survive water temperatures significantly below body temperature for a limited amount of time [39]. An agent who remains on a water patch for a certain number of hours will die of hypothermia. We modeled the effect of hypothermia as a function of time and water temperature (see ODD+D protocol of the model, 10.5281/zenodo.4725959). For the purpose of simplicity, all water patches possess the same water temperature. In the model, the water temperature can be set at intervals of 8°C, which range from 0°C to 39°C. By default, we assume the warm water temperature to be between 24°C and 31°C, which roughly corresponds to temperatures of the Mediterranean Sea that can be expected during a warm phase in the Pleistocene [40].

As the water barrier consists of salt water, agents may die of dehydration. In the literature, the estimated survival time without a supply of freshwater is, on average, 100 hours, ~ 4 days [41]. Thus, we defined the time for the agents’ survival without freshwater as 4 days (96 hours), as default.

Agents who survive the movement on water patches may arrive at a land patch. Upon arrival, their energy storage is replenished to its maximum value of 45,000 energy-units, irrespective of the resource quality on the patch, while the level of dehydration is reset to 0. Aside from dying, the agents may also leave the map. This occurs when an agent moves on a patch at the border of the map, either by a movement decision or by deflection by the water current. Agents who die or leave the map are subsequently replaced in corresponding numbers; new agents appear on a random patch in the source area. Consequently, the total number of agents remains constant (n = 1,000).

### Design of the experiments

As a tool of analysis, we monitor the CSR to quantify how successful agents have been in crossing a water barrier. A certain number of agents (a threshold) reaching the opposite shore is required before the crossing counts as being successful. A founder group consists of a minimum number of agents (n = 50) who are simultaneously present at the opposite shore. The success rate in crossing a water barrier (CSR) is determined as:

$$CSR=\sum_{t=1}^{n}\frac{f_{t}}{a_{t}}$$ (2),

where *CSR* is the crossing success rate after *n* hours, *f*_*t*_ is the number of times a founder group is established and *a*_*t*_ is the cumulative total number of attempts of water crossing. The number of attempts to cross the water barrier correspond to the total number of agents who move from a land patch of the source area to a water patch divided by the founder group size. A simulation time step (“tick”) represents one hour. We ran each simulation experiment for 90 days (= 2,160 ticks).

Here, we refer to the concept of a founder population following Mayr [42–44]. In the literature, estimates for the lower threshold for reported founder population sizes among humans range from 15 [45] to 100 individuals [46]. By default, the minimum number of founder agents is set to an intermediate level of 50. Agents who have already reached the target area may also return to the source area by random movement, therefore, the repeated arrivals of an individual agent in the target area will only be counted once, when the agent arrives for the first time, in order to avoid “double-counting”.

CSR represents a proportional value and varies between 0 and 1. If the CSR is 0, no founder group is established during the simulation run. Although some agents may have accomplished the crossing, the required minimum number of agents for a founder group is not reached. Whereas, if CSR is 1, all attempts of crossing the water barrier result in successful founder groups.

### Systematic testing of the model

We set up experiments in order to test the effect of selected model factors together with four different modes of movement in a systematic way.

The model consists of 18 factors that affect the features and behavior of the agents or the environment (Table 2). We selected five factors to be varied for the simulation experiments while the remaining factors are set to the default levels. In this study, we intend to focus on the performance in water crossings.

**Table 2:** Default levels of model factors.

Agent/Environment	Category	Factor	Default level	Spatial effect
*Environment*	*Environmental*	current-speed*	0 km/hour	water
*Environment*	*Environmental*	water-barrier-size*	10 km	water
*Environment*	*Environmental*	water-temperature-interval*	24–31°C	water
*Agent behavior*	*Movement*	*movement-in-water-direction*	“random”	water
*Agent behavior*	*Movement*	*movement-in-water-speed*	2 km/hour	water
*Agent behavior*	*Movement*	*optimal-walking-speed*	5 km/hour	land
*Agent behavior*	*Perception*	decision-randomness*	0.1	land
*Agent feature*	*Demographics*	death-rate-in-water*	0.0	water
*Agent feature*	*Demographics*	number-founder-agents*	50	land (target area)
*Agent feature*	*Demographics*	population-density*	0.100 individuals/km^2^	land
*Agent feature*	*Physiology*	basal-metabolic-rate-per-day*	1500 energy-units/day	water
*Agent feature*	*Physiology*	*dehydration*	On	water
*Agent feature*	*Physiology*	*energy-loss-water-movement*	800 energy-units/hour	water
*Agent feature*	*Physiology*	hours-until-dehydration*	96 hours	water
*Agent feature*	*Physiology*	*hypothermia*	On	water
*Agent feature*	*Physiology*	*max-energy*	45,000 energy-units	total map
*Agent feature*	*Perception*	perception-radius*	20 km	land
*Agent feature*	*Physiology*	*thermoregulation*	On	water

The factors that were varied in the simulation experiments are marked by an asterisk.

As the model contains sources of stochasticity, i.e. a randomized distribution of resource quality, a random initial placement of the agents in the source area and a certain degree of randomness due to the factor ‘randomness of decision’, it is necessary to determine the number of simulation runs required for capturing the variability of the CSR response to ensure statistical validity of the results. For this purpose, we perform a replication assessment with the default levels (Table 2), as described in Hoad et al. [47] and Lorig [48], prior to the simulation experiments. The precision after *n* simulation runs, *d*_*n*_, is based on the estimation of the confidence interval and is defined as:

dn=tn−1,α2⋅SnnXn¯ (3),

where *n* is the number of simulation runs, *t*_*n*−1_,_*α*/2_ is the student t-distribution quantile, *S*_*n*_ is the cumulative standard deviation, and $\overset{-}{X_{n}}$ is the cumulative mean. We note that a wider confidence interval would lead to more accurate results, although this would, in many cases, massively increase the required number of simulation runs. Thus, in order to balance the statistical validity and simulation performance, we decided that a 95% confidence interval would be sufficient for the simulation experiments conducted in this study. After determining the required number of simulation runs, we conduct one-factor and two-factor sensitivity experiments. In the one-factor sensitivity experiments, we vary one factor while all other factors remain at the default levels and, in the two-factor sensitivity experiment, we vary two factors in combination while keeping the other factors at default levels. All simulation experiments that were conducted for this study are listed in the S3 File. While the one-factor sensitivity experiments focus on the impact of individual factors, the two-factor experiment permits the identification of systematic interactions between two factors. In this study, we categorize the 18 factors into four categories (Table 2). In the first category, we summarize the environmental factors of the model; these modulate the configuration of the water barrier in terms of its extent (*water-barrier-size*), speed of the current (*current-speed*) and temperature of the water (*water-temperature-interval*). In the second category, we summarize the factors of perception; these determine to what extent the agents can assess the resource qualities (*decision-randomness*) and perceive their environment (*perception-radius*). In the third category, we summarize the factors that determine the physiological conditions of the agents in terms of dehydration (*dehydration* and *hours-until-dehydration*), exhaustion (*basal-metabolic-rate*, *energy-loss-water-movement*, *max-energy* and *thermoregulation*) and hypothermia (*hypothermia*) when moving in the water. In the fourth category, we summarize the demographic properties of the founder group (*number-founder-agents*), the number of agents on the map (*population-density*), and the random death rate in the water (*death-rate-in-water*).

The factor levels of the sensitivity experiments are listed in Table 3. Firstly, we vary the extent of the water barrier (*water-barrier-size)* to test the distances being crossed in a particular behavioral scenario, by varying this factor, we may estimate to what extent a water barrier of a certain size impedes successful crossings. Secondly, we vary the radius of perception (*perception-radius*) to test different distances for perceiving the resource qualities, by varying this factor, we may estimate the effect of reduced or increased distances of perception. Thirdly, we vary the hours until dehydration (*hours-until-dehydration*) to test different durations of survival without drinking water while trying to cross the water barrier, by varying this factor, we may estimate the effect of a reduced or increased time of survival without drinking water on the crossing success. Fourthly, we vary the number of founder agents (*number-of-founder-agents*) to test various thresholds for the number of agents required to be simultaneously present in the target area for the crossing to be rated as being successful. Thus, we are able to quantify the decrease of crossing success along with increasing numbers of founder agents, and the increase in crossing success with decreasing numbers of founder agents.

**Table 3:** Factor levels of sensitivity experiments.

Factor	Level 1	Level 2	Level 3	Level 4	Level 5
water-barrier-size*	5	10	15	20	25
*decision-randomness*	0	0.1	0.2	0.3	0.4
perception-radius*	10	15	20	25	30
*hours-until-dehydration*	48	72	96	120	144
*number-founder-agents*	30	40	50	60	70

Default levels are shaded in gray. Factors with which we also conducted the two-factor sensitivity experiments are marked with an asterisk.

Finally, we vary the radius of perception (*perception-radius*) and extent of the water barrier (*water-barrier-size*) simultaneously to analyze the combined effects between different ranges of the perception of resource qualities and different distances between the source- and target shores (Table 3).

Additional factors which are not relevant for the simulation experiments presented in this study, are to be found in the ODD+D protocol of the model which is available online (10.5281/zenodo.4725959).

### Behavioral scenarios

The model allows representing a variety of behavioral scenarios by configuring the direction of movement in the water (*movement-in-water-*direction), the speed (*water-movement-*speed), energy loss (*energy-loss-*swimming), hypothermia (*hypothermia*) and thermoregulation (*thermoregulation*). We test four behavioral scenarios that represent different water movement skills concerning water crossing performance and navigation, ranging from randomly directed paddling and drifting to controlled swimming and rafting.

The scenarios only differ by the water-bound movement (Table 4).

**Table 4:** The four different behavioral scenarios tested in the simulation experiments and their respective levels of factors.

Scenario	A	B	C	D
*movement-in-water-direction*	“random”	“random”	“directed”	“directed”
*water-movement-speed*	2	2	2	3
*energy-loss-swimming*	800	0	800	383
*hypothermia*	On	On	On	Off
*thermoregulation*	On	On	On	Off

Scenario A is driven by resource quality and random paddling; Scenario B is driven by resource quality and random drifting; Scenario C is driven by target area and directed swimming and Scenario D is driven by target area and directed rafting.

In Scenario A, which corresponds to the default levels of the factors, the hominin agents move on water patches by undirected paddling (Table 4).

Although water patches possess a resource quality of zero, they may, however, be preferred in a case of random selection or if reaching a perceived target patch requires movement across water. If an agent ends up in a water patch, they will start to paddle or swim. In Scenario A, we assume that the agents have no control of the paddling direction and merely try to avoid drowning. Paddling is the minimum requirement to examine the possibility of water crossings. We implemented this by defining that the agents paddle in a random direction with a constant speed of 2 km/h.

**Fig 3:**
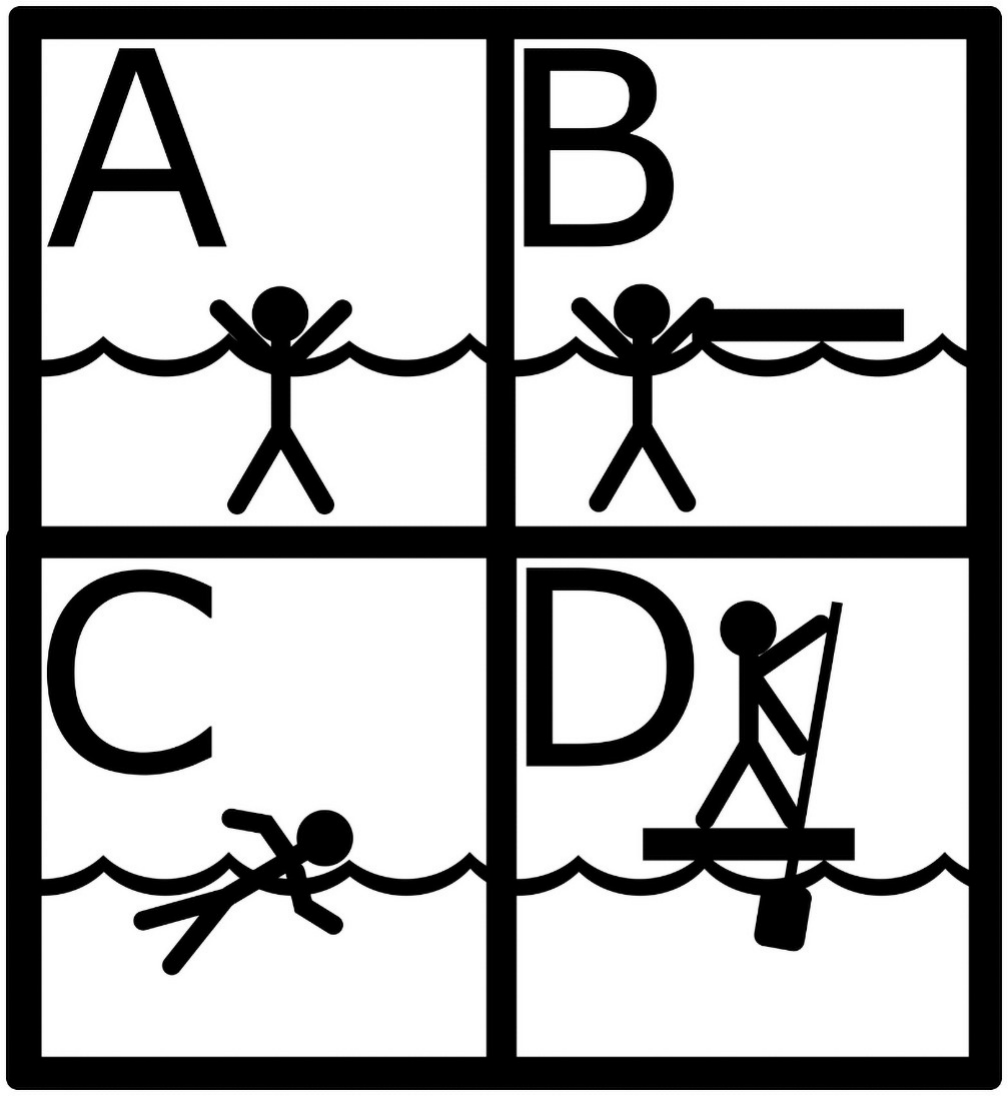
Icons representing the water movement skills in the scenarios. The black bar represents a drifting vessel or a raft. Note that in Scenario B the major part of the agent’s body is under water and is, therefore, affected by hypothermia and thermoregulation.

In Scenario B, the agents move across water patches by drifting. Here, we model the situation where an agent holds on to a drifting piece of wood or vegetation, while the major part of the body remains under water. This scenario corresponds to the situation in which the drifting carrier does not allow the agent to sit on top, because it is too small or not suitable. A drifting carrier functions as a natural life raft. Furthermore, the hominin agent drifts passively and cannot control the direction. When holding on to the drifting carrier, there is no need to perform swimming, therefore, the agent does not lose additional energy besides the basal rate of energy loss and energy loss for thermoregulation. However, as the body remains under water, it is affected by hypothermia and thermoregulation (Fig 3).

In Scenario C, the hominin agents swim directly towards the target area on the opposite shore with a swimming speed of 2 km/h (Table 4). In the same way as in Scenario A, the agents are affected by additional energy loss by swimming.

In Scenario D, the hominin agents use rafts to navigate directly to the opposite shore. Contrary to Scenario B, the agents sit on top of the raft and, therefore, they are not affected by hypothermia. Compared to swimming or drifting, the agents raft with a faster speed of 3 km/h, which is at the lower end of the speed assumed for modern day kayakers [49].

In all scenarios, if the agent has reached the opposite shore, they will then switch to movement on land.

We developed the model in accordance with best practices, such as a standardized terminology [25], replication assessment [47, 48] and, sensitivity studies [50], as well as by providing an ODD+D protocol, which is a standardized protocol for agent-based models [51, 52]. The model was developed in NetLogo 6.1.1. [53], and is available online, along with the ODD+D protocol, at Zenodo (10.5281/zenodo.4725959).

## Results

### Replication assessment

**Fig 4:**
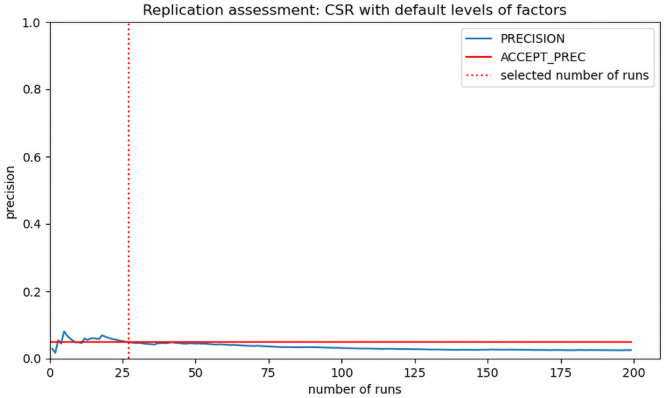
Replication assessment with default levels of factors. The precision is shown by the blue line and indicates the statistical validity of the simulation results at a given number of runs. The threshold for the accepted precision of 5% is shown in red. The number of runs we selected for the simulation experiments and where the precision is consistently below 5% is shown by the dotted line in red.

We performed the replication assessment (see section “Systematic testing of the model”) with the default levels of the factors (Table 3) and 200 runs of the simulation in order to demonstrate that the model is not deterministic and also to identify the required number of runs for capturing the variability in the simulation experiments. In addition, we applied the replication assessment with a 5% significance level with which 27 runs were shown to be consistently below the 5% threshold for precision (Fig 4), thus, we opted for 27 simulation runs in the sensitivity experiments.

### One-factor sensitivity experiments

**Fig 5:**
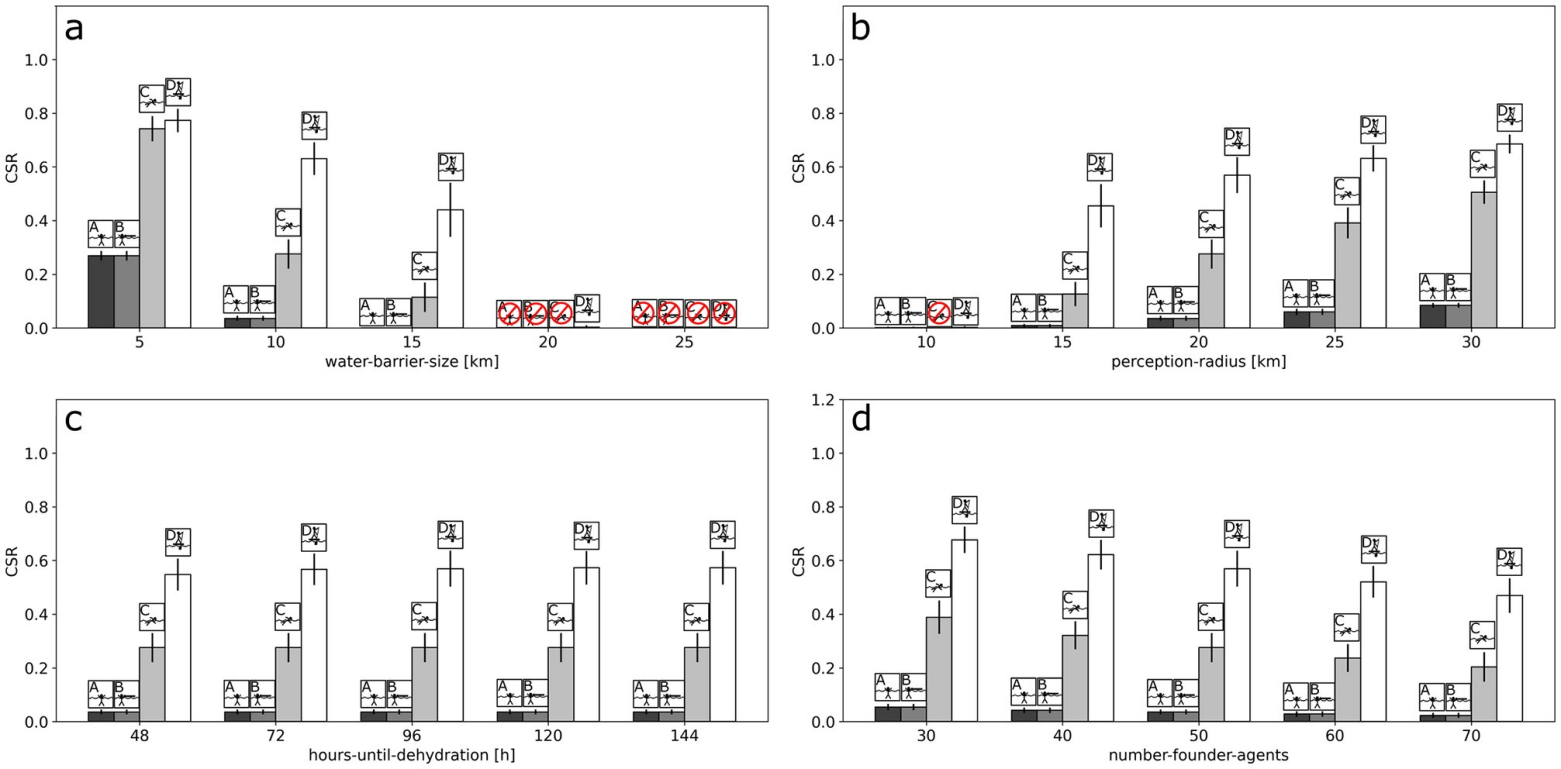
One-factor sensitivity with respect to average CSRs. The one-factor sensitivity experiments show how the systematic variation of levels of a single factor affect the average CSR response. Results are shown for *water-barrier-size* (a), *perception-radius* (b), *hours-until-dehydration* (c) and *number-founder-agents* (d). The behavioral scenarios are represented by the icons. Crossed out red circles indicate no successful crossing (CSR = 0).

The one-factor sensitivity experiments that we conducted consisted of the extent of the water barrier (*water-barrier-*size), radius of perception (*perception-radius*), hours until dehydration (*hours-until-dehydration*) and the number of founder agents (*number-founder-agents*). With our results we are able to demonstrate how these factors affect the CSR (Fig 5).

For the default scenario (Scenario A), increasing the extent of the water barrier decreased the CSR (Fig 5a, Scenario A); its highest average CSR response was at 5 km, with a CSR of ~ 0.27, whilst the responses were close to zero at 10 km (CSR ~ 0.0002) and zero CSR at 15 km onwards. The result was the same for Scenario B (Fig 5a, Scenario B). The directed Scenarios C and D also displayed a decrease (Fig 5a, Scenarios C and D). However, the CSR response was consistently higher up to an extent of 15 km for the water barrier, with the average CSR ranging from ~ 0.11 to 0.44, while reaching zero for Scenario C at 20 km and nearly zero for Scenario D (CSR ~ 0.004). As expected, no successful crossings were achieved at 25 km with any of the four scenarios. On the one hand, this is because the opposite shore cannot be perceived with the default level of perception, which makes deliberate crossings impossible and, on the other hand, the distance makes accidental crossings very unlikely. The relative success of rafting (Scenario D) compared to swimming (Scenario C) also increased with the extent of the water barrier in the directed scenarios.

Increasing the radius of perception was found to increase the CSR in the default scenario (Fig 5b, Scenario A). However, the CSR was close to zero at a radius of perception of 10 km, where the opposite shore is barely visible. This changed substantially at a radius of perception of 15 km, when the opposite shore can be perceived. Here, the default scenario reached a CSR of ~ 0.09. Again, the response of Scenario B was identical to the default (Fig 5b, Scenario B). The directed Scenarios C and D also showed increases in CSR, but reached substantially higher responses, ranging from ~ 0.13 to ~0.45 (Fig 5b, Scenarios C and D). As expected, rafting was found to outperform swimming to a substantial degree in the directed scenarios.

Increasing the hours until dehydration had no effect on CSR in the default scenario (Fig 5c, Scenario A). The same applied to Scenarios B and C (Fig 5c, Scenarios B and C). Only in Scenario D was the CSR slightly decreased at 48 hours of dehydration (CSR ~ 0.55) compared to 72 hours and onwards (CSR ~ 0.57) (Fig 5c, Scenario D). This indicates that dehydration does not play a key role under the levels tested. This is also reflected by the causes for disappearance (see S1 File), where no case of dehydration occurred within Scenarios A-C and only a few with Scenario D.

The CSR in the default scenario decreased, when increasing the number of agents required for establishing a founder group (Fig 5d, Scenario A). The same can be observed in the remaining scenarios (Fig 5d, Scenarios B, C and D). Founder groups may, nonetheless, be established in all of the four scenarios, even if 70 agents are required. Moreover, there were no substantial differences in the CSRs within the undirected scenarios, whereas in the directed scenarios, a relative difference of almost twice as much success between Scenarios C and D persisted throughout the levels.

### Two-factor sensitivity experiment

**Fig 6:**
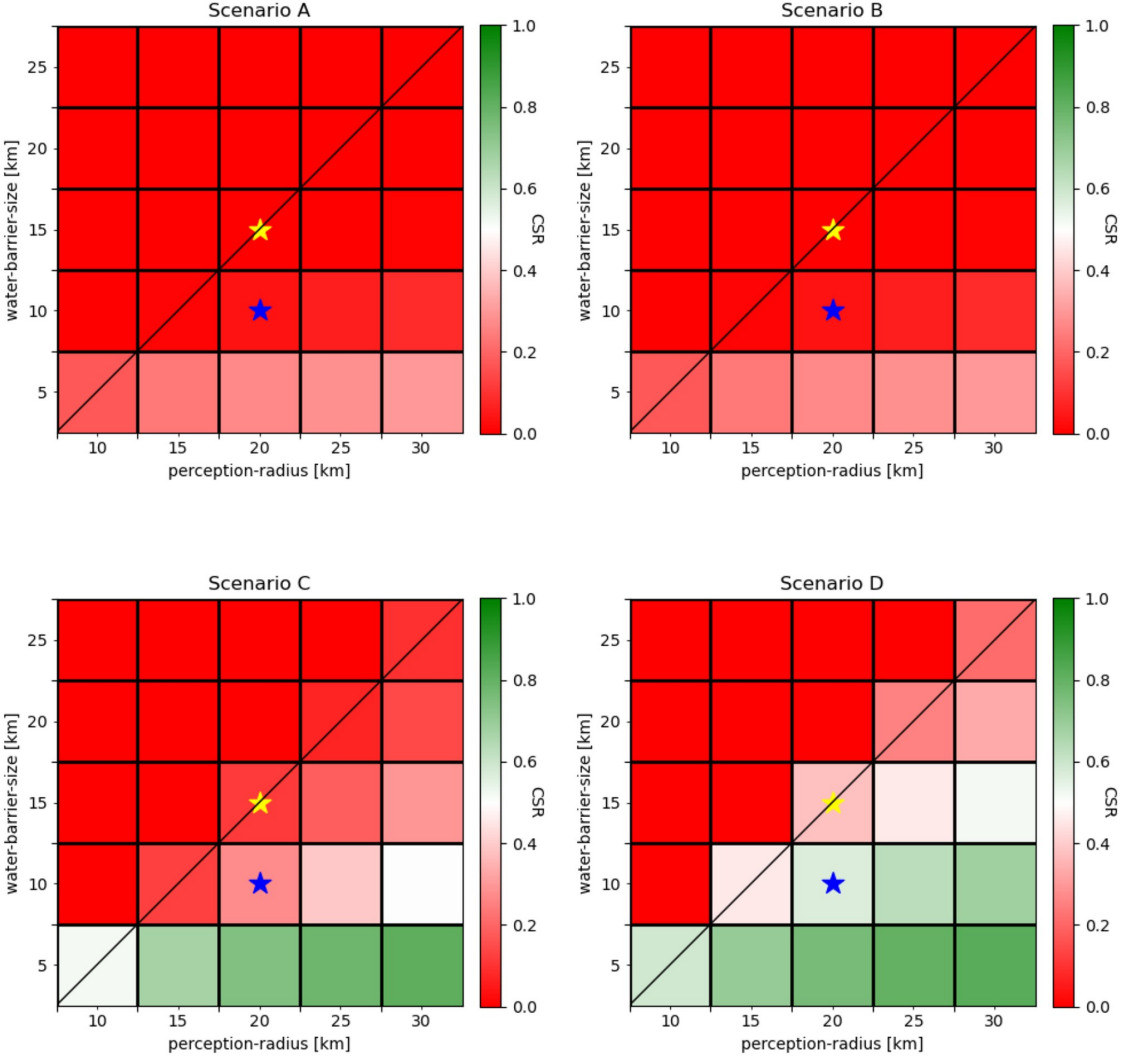
Two-factor sensitivity experiments for the radius of perception (perception-radius) and extent of the water barrier (water-barrier-size) for each scenario. The two-factor sensitivity experiments show the combined effect of the systematic and simultaneous variation of two factors on the average CSR response. The default response is shown by the blue asterisk and the median response is shown by the yellow asterisk. The position of the yellow asterisk in relation to the diagonal line displays the weight of the contribution of each of the two factors.

In the two-factor sensitivity experiment, we varied the radius of perception against the extent of the water barrier. Again, the major difference in the CSR is between the undirected Scenarios A and B, and the directed Scenarios C and D (Figs 5 and 6).

As expected, the highest CSR was achieved when the radius of perception was high and the extent of the water barrier was low. The directed scenarios also achieved substantially higher CSRs than the undirected scenarios. In the experiments, the highest CSRs were accomplished with a radius of perception of 30 km and an extent of the water barrier of 5 km, while CSRs of zero were achieved at a radius of perception of 10–15 km and an extent of the water barrier of 20–25 km. There was no substantial difference among the undirected scenarios, however, in the directed scenarios, Scenario D accomplished a considerably higher CSR than Scenario C. As intended, no successful crossings could be accomplished in the directed scenarios when the extent of the water barrier exceeded the radius of perception. In fact, perceiving the opposite shore and the extent of the water barrier equally contributed to the overall crossing success (Fig 6).

## Discussion

We introduced the CSR as a response to compare the significance of various factors in the model and the performances of different behavioral scenarios on different landscape configurations in a quantitative manner. By means of the CSR, we could show which environmental and behavioral factors contributed to the successful establishment of a minimum number of agents at the opposite shore. The resulting value of the CSR depends on the number of crossing attempts and the number of times a founder group is established at the opposite shore within the given period of 90 days.

### Sensitivity studies

We tested the functionality of the model by systematically varying either one or two factor levels and monitoring the CSR response in comparison to the default levels of the factors (Table 2).

#### Environmental factors

**Fig 7:**
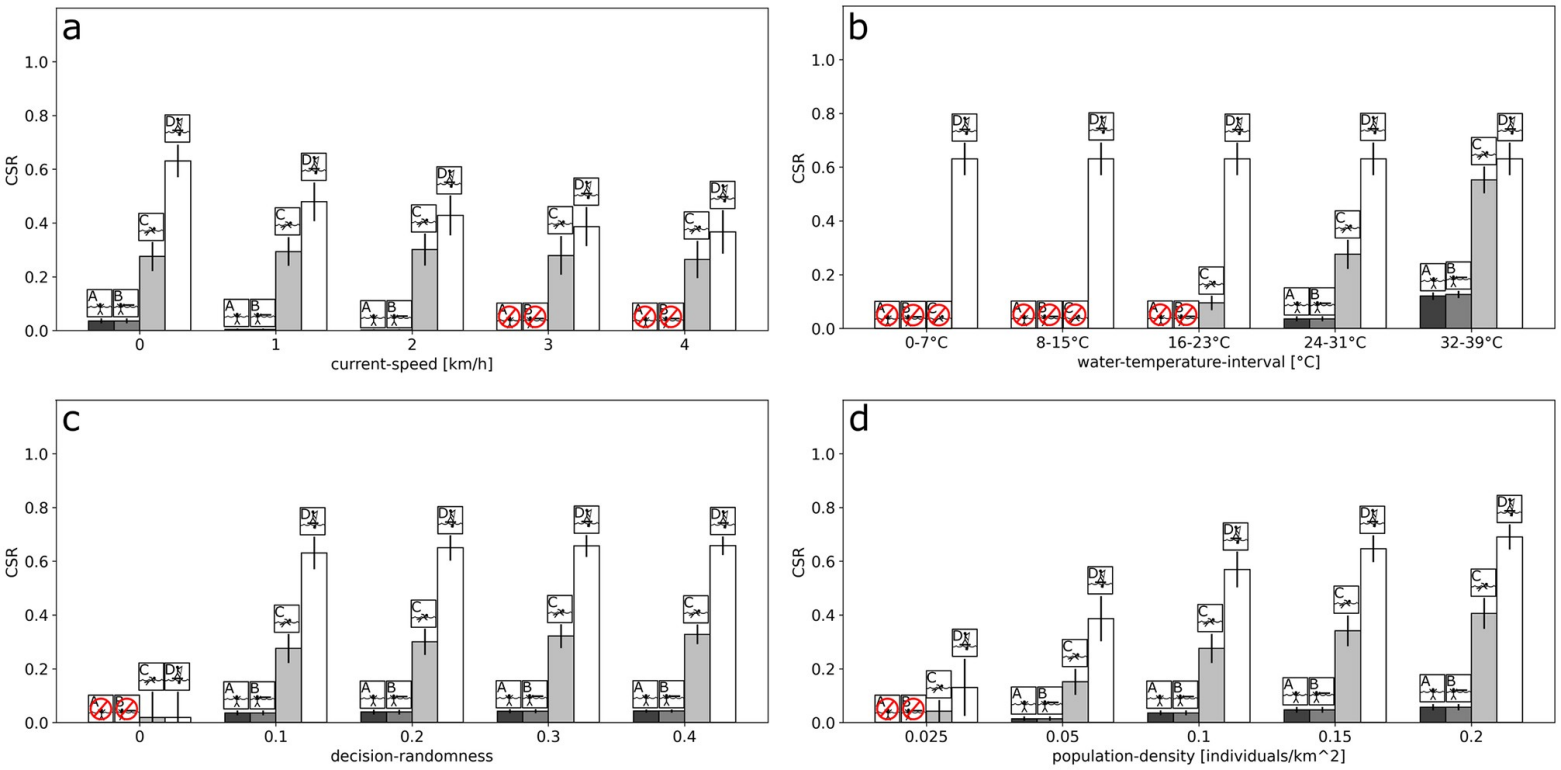
One-factor sensitivity with respect to average CSRs. The one-factor sensitivity experiments show how the systematic variation of the levels of a single factor affect the average CSR response. Results are shown for current-speed (a), water-temperature-interval (b), decision-randomness (c) and population-density (d). The behavioral scenarios are represented by the icons. Crossed out red circles indicate no successful crossing (CSR = 0).

Our model allows configuring of the properties of the water barrier, i.e., its extent, the speed of a potentially deflecting ocean current and the temperature of the water. We selected the extent of the water barrier to be presented in the results section because it is discussed as a limiting factor for sea crossings [4, 7, 54, 55]. With the example of the extent of the water barrier, we show how properties of the water barrier affect the success in crossing. A larger extent of the water barrier reduces the chance for successful crossings. For the case of the directed Scenarios C and D, our results imply that distances of up to 15 km can be crossed with a high chance of success. This is roughly the distance that would be needed to cross the Gibraltar Strait [56–58], and would also be sufficient for crossing the Bab-al-Mandab Strait via island hopping at low sea level [59]. The critical distance is determined by the perception of the opposite shore (discussed in further detail in the following section). However, for larger distances exceeding any conceivable range of perception, for example, the distance of 140 km required for crossing the Sicily Strait [60], further testing would be necessary. Nonetheless, our results suggest that for distances of up to 15 km, additional factors are required to prevent a directed crossing attempt by swimming or rafting. For instance, increasing the speed of the current in the model (Fig 7a) leads to a decrease in crossing success in the directed Scenarios C and D, because this increases the amount of time the agents spend in the water. Correspondingly, the agents have a higher chance of being washed off the map. Lowering the temperature of the water also substantially decreases crossing success by swimming (Fig 7b).

In the artificial model presented here, the speed of the current and the temperature of the water are represented in a simplified and uniform manner. In reality, the speed and direction of ocean currents, as well as water temperature, vary across a sea strait and with geographical regions, seasons and climatic stages [61–63]. When applied to a specific geography, the environmental model may be extended by more differentiated sea surface temperatures [64] and models of oceanic surface currents [61, 65]. Nonetheless, our model allows one to define uniform configurations of currents and temperature to approximate the average effects on crossing success.

In conclusion, the environmental factors in our model allow the adapting of a set of configurations of the water barrier to approximate the properties of certain sea straits in conjunction with climatic conditions, from which the effects on the crossing success can be tested quantitatively.

#### Factors of perception

The perception of the environment determines the directionality of movement. In the model, the perception is determined by the radius of perception and the randomness of decisions. The two-factor sensitivity experiment illustrates the contribution of each factor to the success in crossing (Fig 6).

The increase in CSR observed with increasing the radius of perception (Fig 5b) indicates that as more of the opposite shore is perceived, the higher is the chance of successfully crossing the water barrier. This is because with a higher radius of perception, more agents will try a crossing, what therefore increases the chance for establishing a founder population on the opposite shore. Here, the radius of perception only affects the crossing decision and not the movement in the water. Future versions of the model should also consider effects of perception during a sea crossing. In particular, inter-visibility of the opposite shore increases the probability of successful directed crossings since drifting from the original course can be compensated more easily due to the target remaining visible [59, 66].

The radius of perception represents the feature of the agent complementing corresponding features of the environment, particularly the extent of the water barrier. The results of the two-factor sensitivity study, in which we varied the radius of perception along with the extent of the water barrier, illustrate that the perception of the opposite shore is a prerequisite for directed crossing attempts (Fig 6). In fact, accidental crossings also become more likely when the opposite shore is perceived. In the two-factor sensitivity experiments, we have demonstrated how the perception of the environment affects the success in crossing. Besides representing the perception by viewed distances, the perception may be extended by shared perceptions present in culturally complex hominin groups; these include the ability to communicate knowledge on the environment or to interpret indirect clues of distant land beyond mere visibility, such as migrating birds, smoke from forest fires [8] or cloud formations and the reflections of shallow lagoons on clouds [67].

The factor for randomness of decisions allows for movements beyond places of optimal resource qualities and instead, targets less optimal places, thereby, the agents avoid being confined to a local optimum. A low degree of randomness prevents the agents from moving towards the opposite shore, while a high degree of randomness increases the chance of this movement. In fact, the results from the directed Scenarios C and D highlight that a degree in randomness of 10% is sufficient for successful crossings, whereas further increasing randomness has no additional effect (Fig 7c).

#### Physiological factors

Physiological demands constrain the survival time of a hominin without access to food sources, which is the case when moving in water. In our model, the physiology of the agents is configured by the level of hydration (*dehydration and hours-until-dehydration*), exhaustion (*basal-metabolic-rate*, *energy-loss-water-movement*, *max-energy* and *thermoregulation*) and hypothermia (*hypothermia*). In Scenarios A-C the agents are not affected by the hours until dehydration because they die of hypothermia before dehydration even comes into play (S1 File). Hypothermia should be considered particularly when crossing the Mediterranean, where sea surface temperatures may drop substantially below 20°C during cold phases [40]. However, the deaths caused by dehydration in Scenario D (S1 File) indicate that it becomes relevant when hypothermia is already avoided as would be the case when staying or sitting on top of a raft where the body is kept dry. Dehydration is less relevant for crossings of only a few hours or a day’s duration, which is sufficient for crossing the Gibraltar Strait or the Bab-al-Mandab Strait. In contrast, for prolonged sea voyages on a rafting device, the level of hydration becomes more relevant and should not be overlooked. A hominin who is supplied with sufficient amounts of freshwater would survive a longer period in the water than a hominin who did not drink for a longer time. In addition, dehydration accelerates with increased physical activity such as swimming and/or heat exposure [41]. Technologies such as gourds, in which a supply of freshwater can be transported, would also increase the time in which one survives while crossing a water barrier. Although these factors are not explicitly represented in the model, their effects may be considered by increasing or decreasing the level of dehydration, respectively.

The insensitivity of the basal metabolic rate within the levels of 1,000–3,000 energy-units/day indicates that this factor may be neglected under conditions resembling the default levels presented in this model (S5 Fig). However, as is the case with the level of hydration, the basal metabolic rate may become more relevant for prolonged sea voyages of modern humans, when supply of both food and freshwater is limited. Here, a lower basal metabolic rate would allow for longer journeys than a higher rate. When the model is applied on crossing larger water barriers, such as the Sicily Strait, the basal metabolic rate may, therefore, be considered as a limiting factor when hypothermia and dehydration are already avoided.

We did not consider individual variations in the physiological composition of the agents due to body composition, age and sex, which would affect the costs of thermoregulation [68], for example. However, the physiological factor levels presented in this study reflect the average values and, thus, allow the approximation of the general effects of the physiological conditions of the agents. The physiological conditions of the agents may be differentiated further when the model is adjusted to structured groups of hominins. This also applies to energy expenditure during rafting, where we assumed the levels of competitive rowers [38]. However, competitive rowers are trained athletes using modern boats which allows for the more efficient use of energy expenditure than what we would expect from a simple raft, thus, further bioenergetic experiments would be necessary to re-calibrate the energy expenditure when using simple rafts. The results from these experiments would then be able to be incorporated into our model to evaluate the effect of increased energy expenditure on crossing success.

#### Demographic factors

Demographic requirements determine the likelihood that a founder population may establish on the opposite shore. In our model, the demographic factors are represented by the density of the population (*population-density*) and the number of individuals required for a founder group (*number-founder-agents*). Our model allows to test the performance of different founder group sizes. Our results suggest that a larger founder group is less likely to be established than a smaller founder group. We note that for a permanent occupation, such a group must not only be able to survive the water crossing, but also to exploit the resources in the new area and reproduce. Therefore, in addition to the mere number of individuals in the group, the composition of the group with regards to the ability to thrive in the new environment needs to be considered. This limits a successful founder group to consist of a sufficient number of reproducible adults. The group also has to adapt to the new environment of the opposite shore, i.e., by adjusting foraging strategies to different types of resources and properties of the landscape.

In our model, higher crossing success can also be achieved by increasing the density of the population (Fig 7d). This is surprising because the chance of an individual agent failing the crossing does not depend on the population density. However, when the population density is high, the rate of agents attempting the crossing is also increased. The agents on the opposite shore, thus, have a shorter time frame for leaving the map without being assigned to a founder group than at a low population density, where the total number of attempts is lower. Therefore, when the model is applied to a particular hominin population, its population density estimate is required for assessing the CSR. The population densities we tested in the model are in the range of the estimated population densities for hominins, but do not include the full range of potential values from 0.001 to 2.48 individuals per square kilometer [33]. In future experiments, the effect of different population densities, which are assumed for specific hominin species, could be compared by means of crossing success. Further factors determining the arrival success in the target area are discussed in more detail below.

The population structure in the model is deliberately kept at a basic level with homogeneous agents. Nevertheless, the model can be extended for questions that address the influence of population structure on crossing success, for example, by differentiating age structures, proportions of subsistence strategies and/or reproduction.

The strength of the CSR is that it combines the outcomes of complex movement decisions into a single value. The CSR may be applied to various geographies and permits one to assess the potential of particular landscapes to function as corridors and/or barriers in the context of hominin expansions.

The CSR serves as a linking interface between small-scale mobility models and large-scale hominin dispersal models. We note that upscaling a small-scale model to a large-scale model is challenging because both types of models consider different types of movement. In small-scale models, movement is expressed by mobility decisions performed by individuals or small groups [69–71], while large-scale models consider movement as a dispersal, which is induced by population growth [22–24, 72]. Both kinds of movement are obviously related, but this relation is not reflected by the modeling approaches. The CSR bridges this conceptual gap.

The challenge in simulating hominin expansions on a large spatio-temporal scale is to balance the behavioral and spatio-temporal complexity [73] as well as the technical constraints due to limited computational power. It is, therefore, probably not desirable, even if it were possible, to construct large scale expansion models at a very detailed and complex level. On the one hand, these models would be too complex to understand, while, on the other hand, limiting computational resources would not allow one to conduct a sufficient number of simulation experiments in any reasonable amount of time. Simulations and models should not be considered as tools to transfer the real world into a computer and an experimental set up [74], instead, these act as abstractions of the real world, representing only a certain aspect of the process under study. Simulation experiments are then conducted to assess the contribution of the aspect in an isolated and controlled setting, before adding another aspect or integrating it into the overarching context. Hence, a different solution is required allowing to synergize the potential from the small- and large-scale perspectives.

By applying CSR as a linking variable, it is possible to complement large-scale hominin expansion models by integrating the small-scale results of particular crossing simulations. The CSR could then be used as an environmental variable controlling the speed of dispersal.

For the simulation of hominin expansions, therefore, we propose the use of an integrative approach that uses small-scale results (such as the CSR) and to integrate these into critical regions of the large-scale model to provide a comprehensive explanation of hominin expansions across different scales.

It is worth noting, that the CSR does not quantify for how long a founder group establishing at the opposite shore is able to survive in the new environment and whether the success in crossing persists across large temporal scales of several thousands of years. Nonetheless, a founder group represents a prerequisite for sustainable settlements at the opposite shore.

Along the same lines, the CSR itself does not characterize particular routes across the water barrier. While this is trivial in a uniform water barrier, as presented in our study, it may be relevant in environments where island hopping is possible and, thus, the agents would have choice of different routes.

### Crossing decision and how to get into the water

The movement on land is deliberately kept at a basic level because the model focuses on the movement in the water. Therefore, the movement on land, as it is implemented in the model, functions as a place holder for more sophisticated mobility and subsistence strategies. In this basic model of movement, the direction is decided upon the resource qualities within the radius of perception and the speed is determined by the slope value. Nonetheless, the land movement in the source area is not monitored by the CSR because the CSR calculation commences with the crossing attempts when the agents move from a land patch, in the source area, to a water patch. Thus, the CSR is only intended to evaluate the crossing of the water barrier and its success in the target area. As already discussed, the agents need to perceive the opposite shore in order to attempt a crossing. Avoiding low water temperatures is crucial because even a difference of a few degrees Celsius severely affects the chance of crossing successfully (Fig 7b).

A crossing attempt may either be deliberately decided upon or may happen by accident, for example, by being swept away while gathering aquatic resources. Deliberate crossing decisions occur because the hominins would expect valuable resources at the opposite shore.

### Crossing the water barrier

#### Paddling and swimming

Within Scenarios A and C, we modeled a basic water movement behavior which is either randomly directed (Scenario A) or is directed towards the target shore (Scenario C). Our results suggest that paddling and swimming should be considered as an option for crossing water barriers of an extent of up to and including 10 km.

The extent of the hominin s’ paddling or swimming performance is not known and is the subject of current research [75]. Even though hominins, such as *Homo erectus* or *Homo floresiensis*, possessed the biomechanical requirements for swimming [6], this appears to be, primarily, a cultural and technical skill [6, 76, 77].

It is well known that, at present, humans can perform long distance swimming, even in open waters, for example, in the context of competitions [78, 79]. An example for a long-distance swim would be swimming across the Gibraltar Strait, where distances of about 14.3 km have to be encountered [56, 80]. The example of the Gibraltar Strait crossings illustrates that crossing a water barrier of several kilometers solely by swimming can be accomplished, at least by modern humans performing specific swimming styles, optimizing energetic needs and speed. This requires extensive preparation and monitoring of the environmental conditions, such as weather and water temperature. The extent to which hominins could plan and prepare for such an endeavor is the subject of current research [81–83].

We wish to note that in our model, the agents do not vary in swimming skill. However, there may be substantial differences in the performance between individuals with regard to prior training, technique and physical fitness [84]. Increasing the stroke rate particularly leads to an increase in swimming speed and additional metabolic heat production [37, 85].

As we do not know the extent of hominin swimming performance, we tested a variety of performance levels. By simulating two different basic water movement performances in Scenarios A and C, we showed that distances of several kilometers would not prevent hominin agents from crossing, even without the use of rafts. Instead, the behavioral difference in the swimming intention has a tremendous impact on the crossing success. The crossing of water barriers should, therefore, be considered as an option in hominin route selection and should be ruled out only if a variety of behavioral models have been tested and failed in crossing the barrier.

#### Drifting and rafting

Analogous to the paddling and swimming performance, we modeled two different levels of maritime transportation devices within the Scenarios B and D. Scenario B represents random movement on water with a drifting carrier, while the majority of the hominin’s body remains under water. In Scenario D, we assume the hominins to remain atop a raft and are in control of the direction (Fig 3).

Our results suggest substantial differences in the crossing successes between using a drifting carrier (default CSR ~ 0.04) and rafting directedly (CSR ~ 0.63).

For Scenario D, the hominins would have required knowledge on how to build basic rafts, for example, from bamboo, bundles of grass or other plant fibers. This would have required them to be able to fasten bundles or sticks together in order to build a floating surface sufficient in size to carry a single person or a group across water. Building such a compound raft would require some degree of collaboration. Moreover, some form of steering technology, such as sticks or paddles is required. Directed rafting probably goes beyond the cognitive skills presumed for hominins during Out-of-Africa 1, but may function as a reference scenario for Middle or Late Pleistocene hominins.

Actualistic experiments in which simple rafts were built with natural materials, such as bamboo, illustrate that distances of up to 80 km could be travelled [86]. When food and freshwater storage technology were available, distances could reach up to several thousands of kilometers [87, 88]. These experiments are, however, restricted to modern humans with Late Pleistocene technology, and it remains to be tested whether simple rafts could be built with earlier technology on the level of Oldowan or Acheulean. In addition, the number of attempts with which these experiments were conducted is low and, furthermore, they were conducted by monitoring climatic conditions and with present day navigational knowledge. Nevertheless, such attempts are valuable because they show that travelling larger distances by simple rafts is possible, in principle.

Therefore, these experiments should be complemented by computer simulations; these would allow simulating attempts of crossing sea straits under various environmental scenarios that resemble conditions during the Pleistocene. By designing appropriate scenarios, our model permits the testing of the whole range of maritime navigation that is presently considered for hominins, ranging from random and passive drifting to directed and controlled rafting. Furthermore, our model permits the design of specific scenarios for other methods of water crossings, such as directed rafting with the body in the water or hitch riding on the back of elephants [89], as well as passive rafting on top of floating vegetation [54, 90, 91], by respective configuration of the factors in Table 4.

#### Undirected vs. directed

The scenarios presented in this study represent two different types of movement in water which may be grouped, one the one hand, into scenarios of movement in random directions (Scenarios A and B) and, on the other, scenarios of movement directed towards the target area on the opposite shore (Scenarios C and D).

Given that Scenarios C and D consistently resulted in considerable higher CSRs than Scenarios A and B, this suggests that the directionality of movement has a major effect on the success of crossing a water barrier.

Furthermore, swimming directly towards the target area (Scenario C) outperformed drifting in a random direction (Scenario B). This indicates that the use of a drifting carrier may not be advantageous if the movement is not directed and that the reduced energy loss due to drifting bears no advantage, at least within favorable conditions and distances of up to and including 10 km. Hence, the use of rafting technology would not be a requirement for crossing such types of water barriers. It is assumed that rafting becomes more important particularly for longer distances exceeding 20–30 km [54]. A more relevant skill would be the monitoring of the environmental conditions that allow for moving directly towards the target area, such as still water and favorable weather, in addition to skills in navigation.

The comparatively shorter time span that one spends in the water while moving directly towards the target area explains the higher success rate in crossing the water barrier because the chances of dying by hypothermia, dehydration or exhaustion is reduced accordingly. Due to the reduced exposure to dangers from the water, successful crossings of larger water barriers become more likely.

Thus, when applying the model, assessing the capabilities of the modeled hominins in performing directed movement towards the target area needs to be carefully considered in order to apply an appropriate skill of movement in the water.

Strong currents may, however, constrain the extent to which a directed movement towards the target area is possible. On the one hand, currents may be utilized as an assisting force. as is observed with fish migration [92–94] or by human triathletes [95], while on the other hand, swimming against the currents would only be possible if the speed of the current is below the speed of swimming.

Although our results suggest that moving directly across the water barrier is substantially more successful in establishing a founder group on the opposite shore, crossings by moving randomly in the water also occurred. We note that, in the real world, the chance of crossing a water barrier accidentally to form a founding population is very low, due to the low probability that individuals can accumulate in sufficient numbers within a short period of time, e.g., months or years.

We may suggest that moving directedly is not a necessity for crossing a water barrier, because if given enough attempts, random movements will lead to reaching the target area sooner or later. Such unintentional water crossings are documented for Iguanidae [96], lizards [97] and primates [98]. Hence, accidental crossings of water barriers need to be considered, especially when the considered time frame would allow for such multiple attempts.

Nonetheless, moving randomly in water without orientation may only occur on rare occasions. These could be, for example, in the context of natural catastrophes, such as tsunamis or hurricanes, or in the water under bad weather conditions, such as fog, high waves or during the dark. Therefore, the simulation runs for the Scenarios A and B, where the agents in the water move in random direction, may overrepresent these rare occasions.

Consequently, when the model is applied for a particular case-study, the chances of environmental conditions which induce loss of orientation need to be assessed and considered.

We note that accidental crossings may also lead to directed crossings. For instance, stranded hominins may emit cues, such as smoke from campfires, or returnees might tell of distant lands. This would, of course, require the ability to interpret indirect signs and communication. For this purpose, the model could be adapted by combining undirected and directed scenarios, as well as integrating complex social interactions, such as collaboration, shared use of rafts and communication. This could be realized by defining groups of individuals as an agent and by allowing agents to exchange information with each other. The same applies for cognitive skills such as monitoring, learning and planning, which permit more complex decisions, such as the cost-benefit assessment of the crossing or the deliberate use of currents. Although agents do not adapt during a simulation run, the behavioral scenarios represent different stages of maritime adaptations, whereat paddling is the lowest stage and rafting the highest. Hence, adaptation rates and their effects on crossing success could be tested by switching between behavioral scenarios during a simulation run. In addition, aquatic resources can be added to the model by assigning resource qualities to the water patches. Agents who are adapted to an aquatic environment and specifically seek aquatic resources will spend more time on the coast, opening up more opportunities for crossing attempts.

### Arrival success in the target area

**Fig 8:**
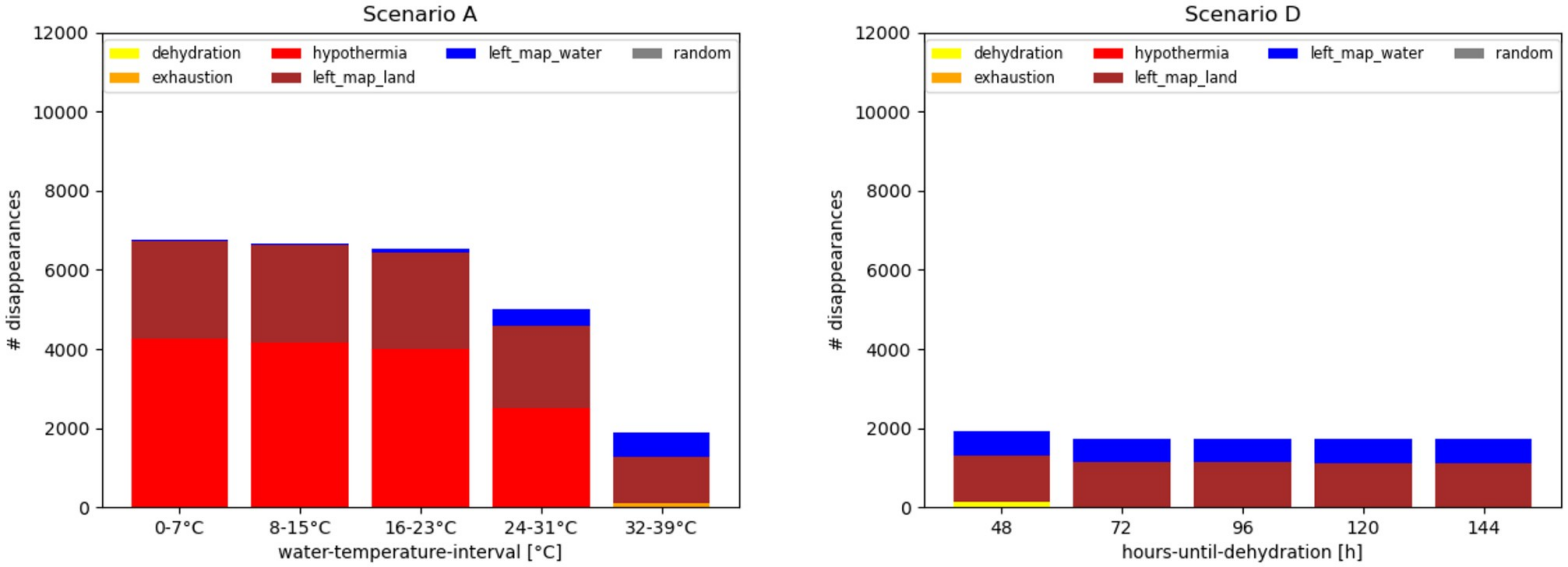
Disappearance of agents during simulation runs. An example of Scenario A, when varying the temperature of the water (left) and an example of Scenario D (right), when varying the hours until dehydration, are shown. Agents may die of dehydration, hypothermia or exhaustion, but may also leave the map at the border across land (left_map_land) or across water (left_map_water).

The success in arriving at the opposite shore is determined by the ability to survive the crossing and the establishment of a viable founder population. In the model, the crossing attempts may fail because agents leave the map or die in the water. When examining the causes of disappearance, the majority of agents disappear by leaving the map on land by moving towards the border of the map, either at the source or the target area. The former case refers to an absence of crossing, whereas the latter case refers to a failed crossing because the agents have already left the target area before a founder population could establish itself. Although some agents may disappear by being washed away by currents, the majority of agents die of hypothermia in Scenarios A-C (see S1 File). Furthermore, death by dehydration can occur, even when hypothermia is avoided, due to a low hydration level, while death by exhaustion occurs when paddling and in warm water temperatures (Fig 8).

The causes of disappearance for all scenarios, are given in the S1 File. For successfully arriving at the opposite shore, a sufficient number of individuals needs to accumulate for establishing a founder group.

As agents may leave the map at the borders, individuals need to accumulate in sufficiently high numbers in the target area before too many of them have left the map. Our simulation experiments show, that when the crossing is directed, such founder populations may establish several times within the time frame of three months under favorable conditions. Although less likely, founder groups may also establish by accident, when crossings are undirected. We may assume that such accidental crossings become more likely when considering extended periods of time, e.g., years or generations, instead of only three months. Populations may establish after several years or generations [99] with preceding two-way crossings [100] that maintain a connection between the source area and the opposite shore, thus, gradually transitioning into viable founder populations. In evolutionary time scales these become even more likely [9]. For applications that address crossings over extended periods of time, the duration of the simulation run can be extended as needed and is only constrained by the computational resources and the available simulation time.

## Conclusion

Hominin expansions are considered as processes that occurred across time periods of millions of years and across continents. Therefore, these are often modeled as large-scale diffusion models [17–20, 22, 23]. However, processes such as the crossing of sea straits occur on much smaller spatio-temporal scales.

Our agent-based model allows one to simulate water crossing with hominin agents that are capable of paddling, drifting, swimming or rafting across a water barrier embedded in artificial landscapes with random environmental variables. As we intended to explore the general effects, we tested five factors in conjunction with four scenarios of skilled performances of movement in water. From the simulations, we can identify circumstances under which water crossing could have been more, or less, likely. We found that the directionality of the movement behavior of the agents along with their correspondingly wide perception have a substantial effect in performing successful and sustainable crossings of sea straits.

This model can act as a template for the simulation and quantifying of the crossing of other landscape features that are assumed to be barriers, such as deserts and/or mountain ranges.

The strength of our model is that it represents the process of water crossing dynamically, by the hominin agents, transitioning from land into water and moving through the water by paddling, drifting, swimming or rafting within rather small spatio-temporal scales. Therefore, future research should intensify the exploration of hominin behavior and develop frameworks with which behavioral models can be integrated and subsequently tested in simulations. Our agent-based model allows formalizing and explicating the numerous conceptual models for hominin sea crossing from archaeology and paleoanthropology (e.g. [2, 11–14]), by configuring the factors of the map environment as well as adjusting the properties of the agents. Simulation then allows the quantitative comparison of the aforementioned conceptual models in terms of successful and/or prevented crossings.

The output variable CSR, with which we quantified the success of the crossing, helps to identify the extent to which environmental behavioral factors contribute to the crossing success. Furthermore, the CSR can be used as an interface, to integrate the results of small-scale processes into large-scale “diffusion-like” hominin expansion models and draws a bridge to the otherwise disconnected scales.

Our model could be applied to reconstructed maps that match with paleoanthropological questions. Moreover, our model can be adapted to provide CSRs for various time periods as well as geographical contexts, for example, hominin expansions out of Africa [101]. Whether hominins expanded into Eurasia across sea straits already during Out-of-Africa 1 [102] is unknown, as there is no direct evidence [103, 104]. However, if they did, then they probably crossed one or more of the sea straits in the Mediterranean or the Red Sea [59], e.g., the Gibraltar Strait, Sicily Strait [60], and/or the Bab-al-Mandab Strait [13], or the Aegean Sea [6, 8, 105]. Further major sea straits of relevance are the Strait of Hormuz [106, 107] as well as island hopping in Wallacea and the Philippines [108]. The same applies to larger rivers such as the Ganges, Indus, Brahmaputra [107, 109]. This would only require to integrate the corresponding GIS data on resources and topography into the model as well as the properties of the water barrier.

Our model can improve existing large-scale agent-based models for hominin sea crossing during the Middle- to Late Pleistocene [92] or modern human sea crossing during the initial expansion into Australia [110], as well as acting as a supplement to least-cost path models for hominin expansions [107, 111, 112].

Our studies show that the model introduced here is responsibly calibrated, thoroughly tested and ready to be applied in geographically explicit analyses. When applied, the simulations will provide valuable insights into the role of sea crossings during hominin expansions.

## Supporting information

S1 Fig One-factor sensitivity for random death rate (death-rate-in-water). The bars show the average CSRs from 27 simulation runs for each factor level.
(TIF)

S2 Fig One-factor sensitivity for dehydration (dehydration). The bars show the average CSRs from 27 simulation runs for each factor level.
(TIF)

S3 Fig One-factor sensitivity for max energy (max-energy). The bars show the average CSRs from 27 simulation runs for each factor level.
(TIF)

S4 Fig One-factor sensitivity for optimal walking speed (optimal-walking-speed). The bars show the average CSRs from 27 simulation runs for each factor level.
(TIF)

S5 Fig One-factor sensitivity for basal metabolic rate (basal-metabolic-rate). The bars show the average CSRs from 27 simulation runs for each factor level.
(TIF)

S1 File Average causes for disappearance of agents in all of four scenarios. The bars show the average number of deaths from 27 simulation runs for each factor level. Agents may disappear by leaving the map or when dying.
(PDF)

S2 File NeLogo outputs. CSV output of the simulation experiments presented in this paper and Python scripts used for creating the graphs.
(ZIP)

S3 File Design points. Simulation settings of all simulation runs that were conducted in this study with their respective Figure references.
(XLSX)
