# Correction: Discovering the opposite shore: How did hominins cross sea straits?

**DOI:** 10.1371/journal.pone.0259484

**Published:** 2021-10-28

**Authors:** Ericson Hölzchen, Christine Hertler, Ana Mateos, Jesús Rodríguez, Jan Ole Berndt, Ingo J. Timm

The ORCID iDs are missing for the second, fifth, and sixth authors. Please see the authors’ respective ORCID iDs here:

Author Christine Hertler’s ORCID iD is: 0000-0002-8252-9674 (https://orcid.org/0000-0002-8252-9674).

Author Jan Ole Berndt’s ORCID iD is: 0000-0001-7241-3291 (https://orcid.org/0000-0001-7241-3291).

Author Ingo J. Timm’s ORCID iD is: 0000-0002-3369-813X (https://orcid.org/0000-0002-3369-813X).
