# Supplementary figures and images for: Discovering the opposite shore: How did hominins cross sea straits?
Source: PLoS One. 2021 Jun 30;16(6):e0252885. doi: 10.1371/journal.pone.0252885 (PMC8244915; doi:10.1371/journal.pone.0252885)

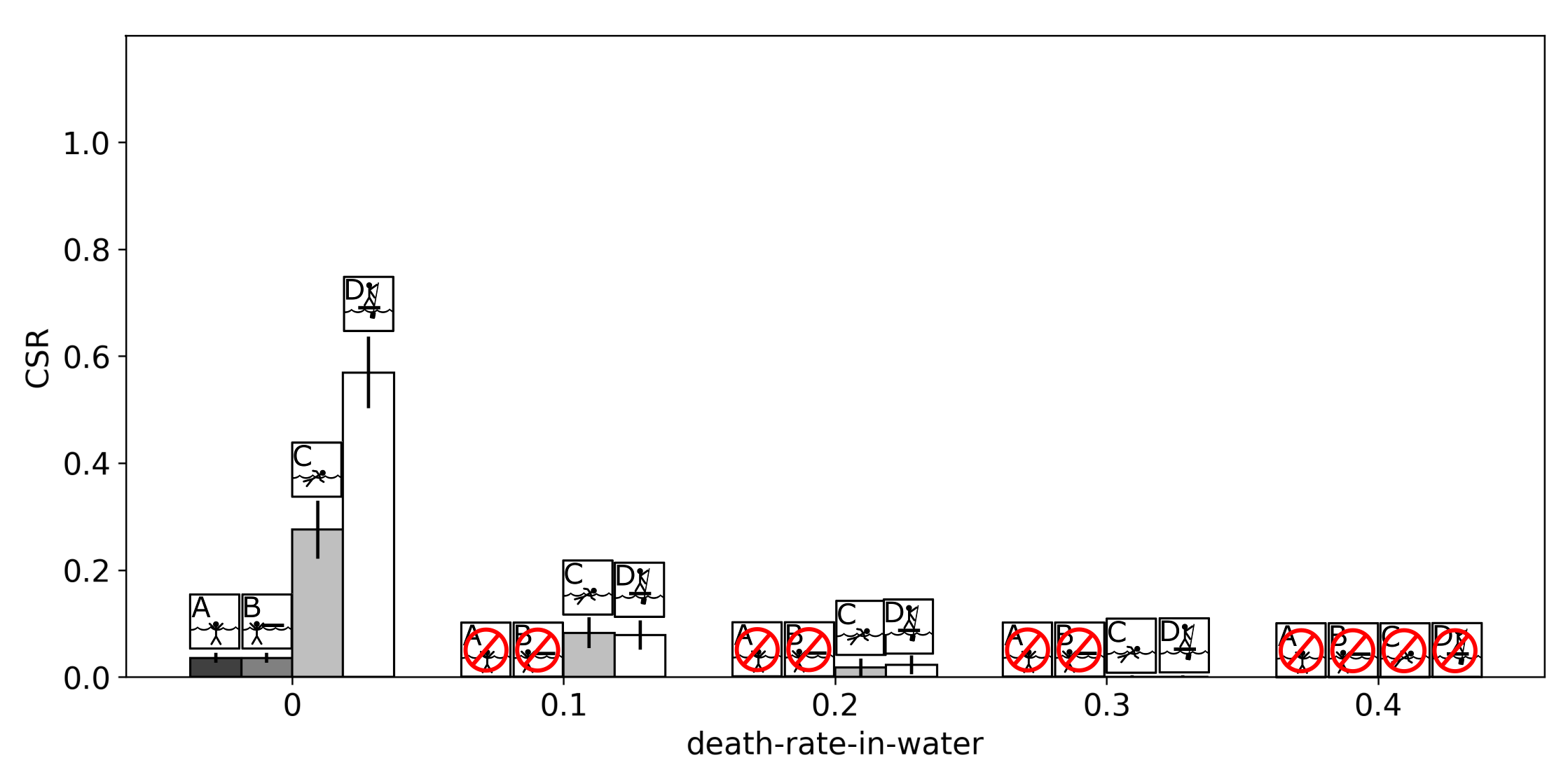

Supplement: S1 Fig — The bars show the average CSRs from 27 simulation runs for each factor level. (TIF) [file pone.0252885.s001.tif]

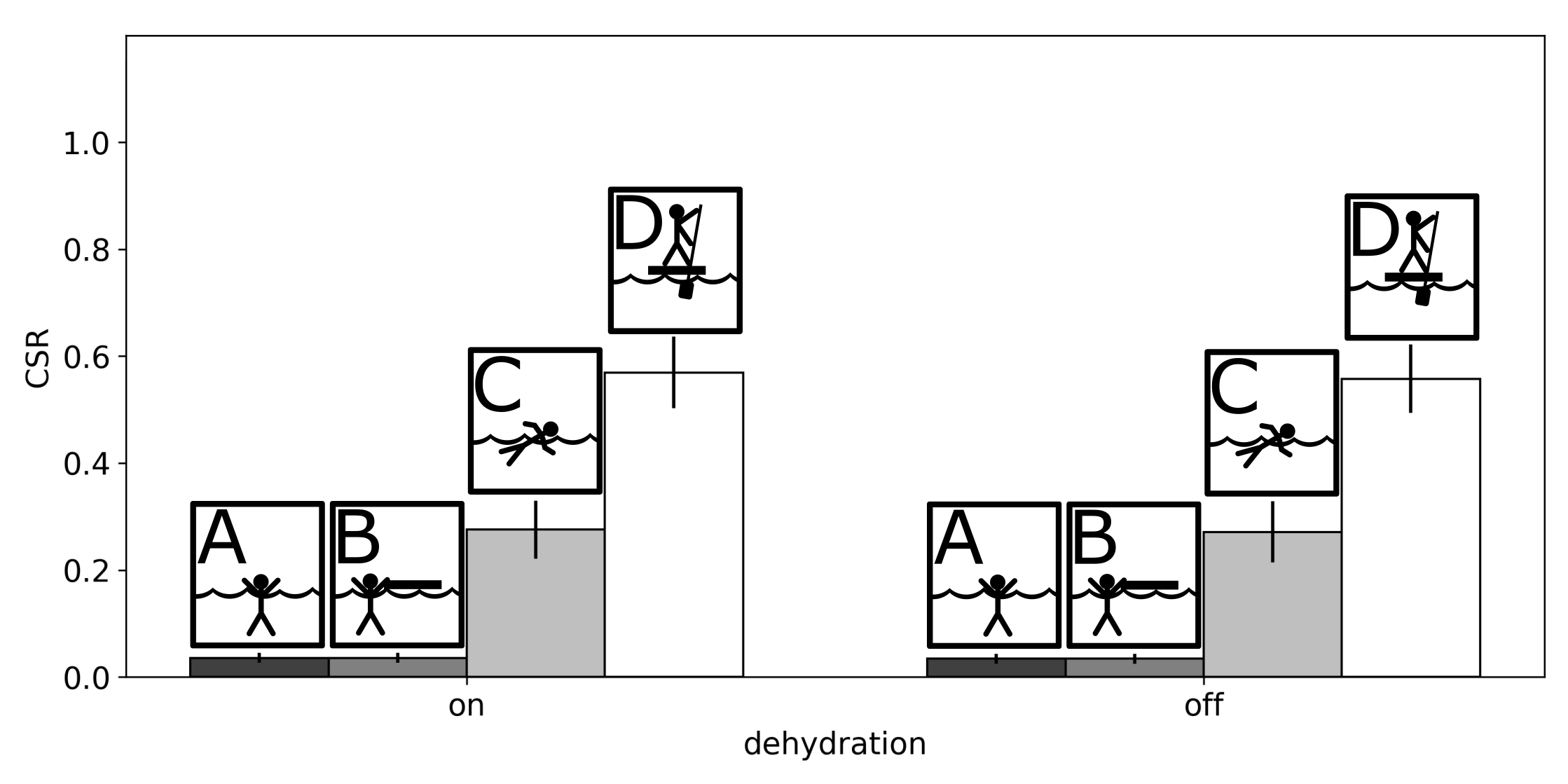

Supplement: S2 Fig — The bars show the average CSRs from 27 simulation runs for each factor level. (TIF) [file pone.0252885.s002.tif]

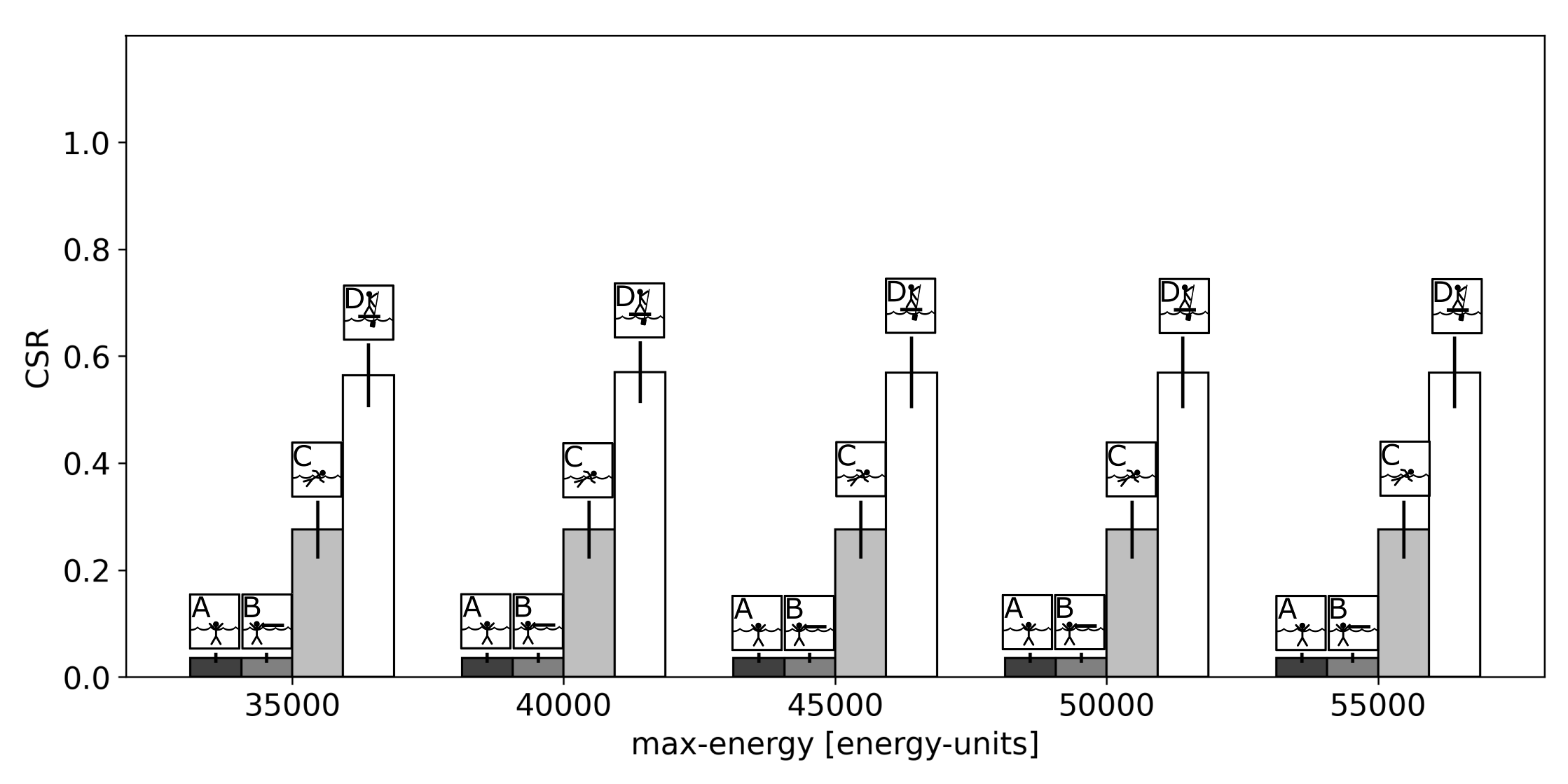

Supplement: S3 Fig — The bars show the average CSRs from 27 simulation runs for each factor level. (TIF) [file pone.0252885.s003.tif]

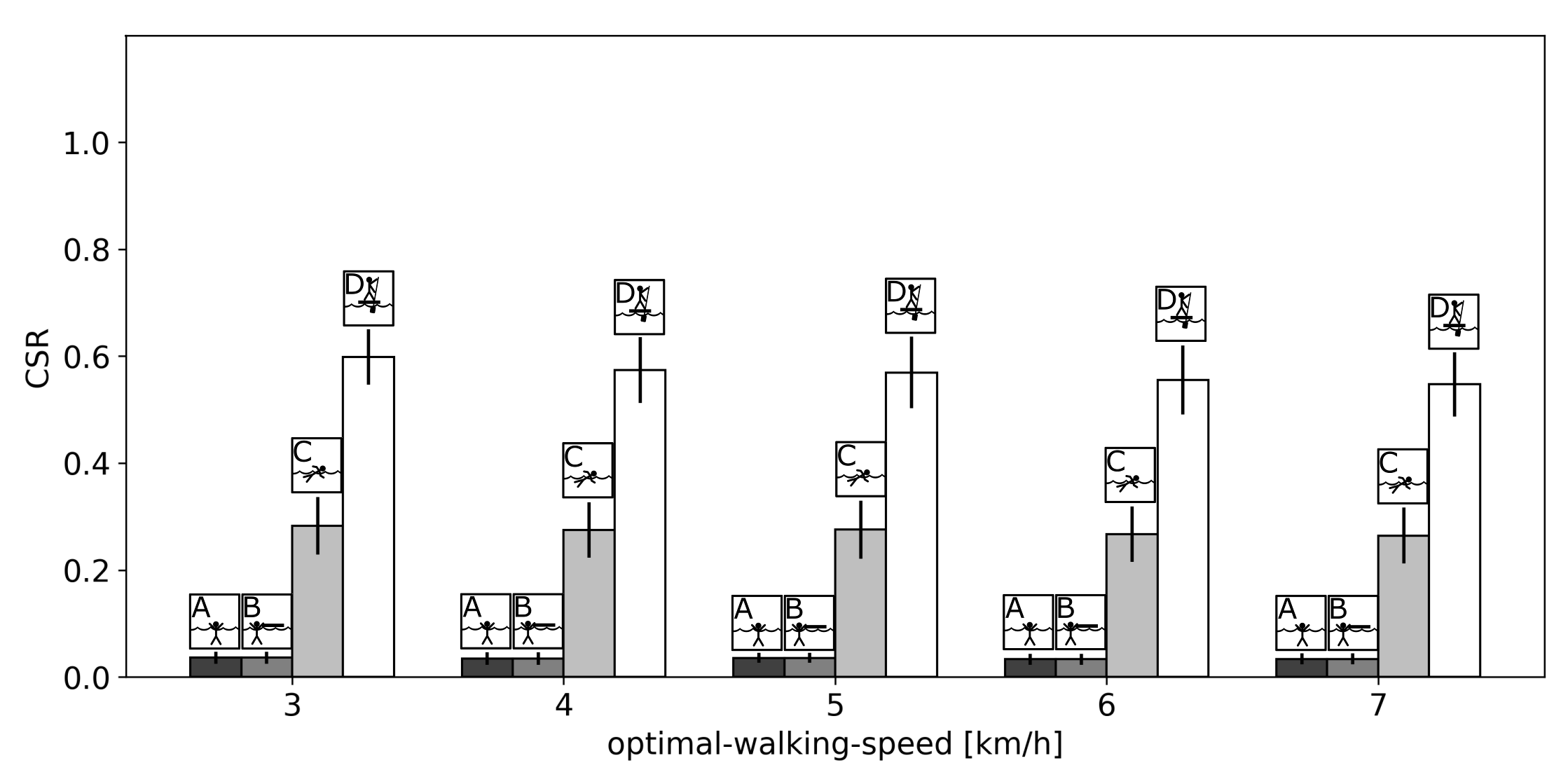

Supplement: S4 Fig — The bars show the average CSRs from 27 simulation runs for each factor level. (TIF) [file pone.0252885.s004.tif]

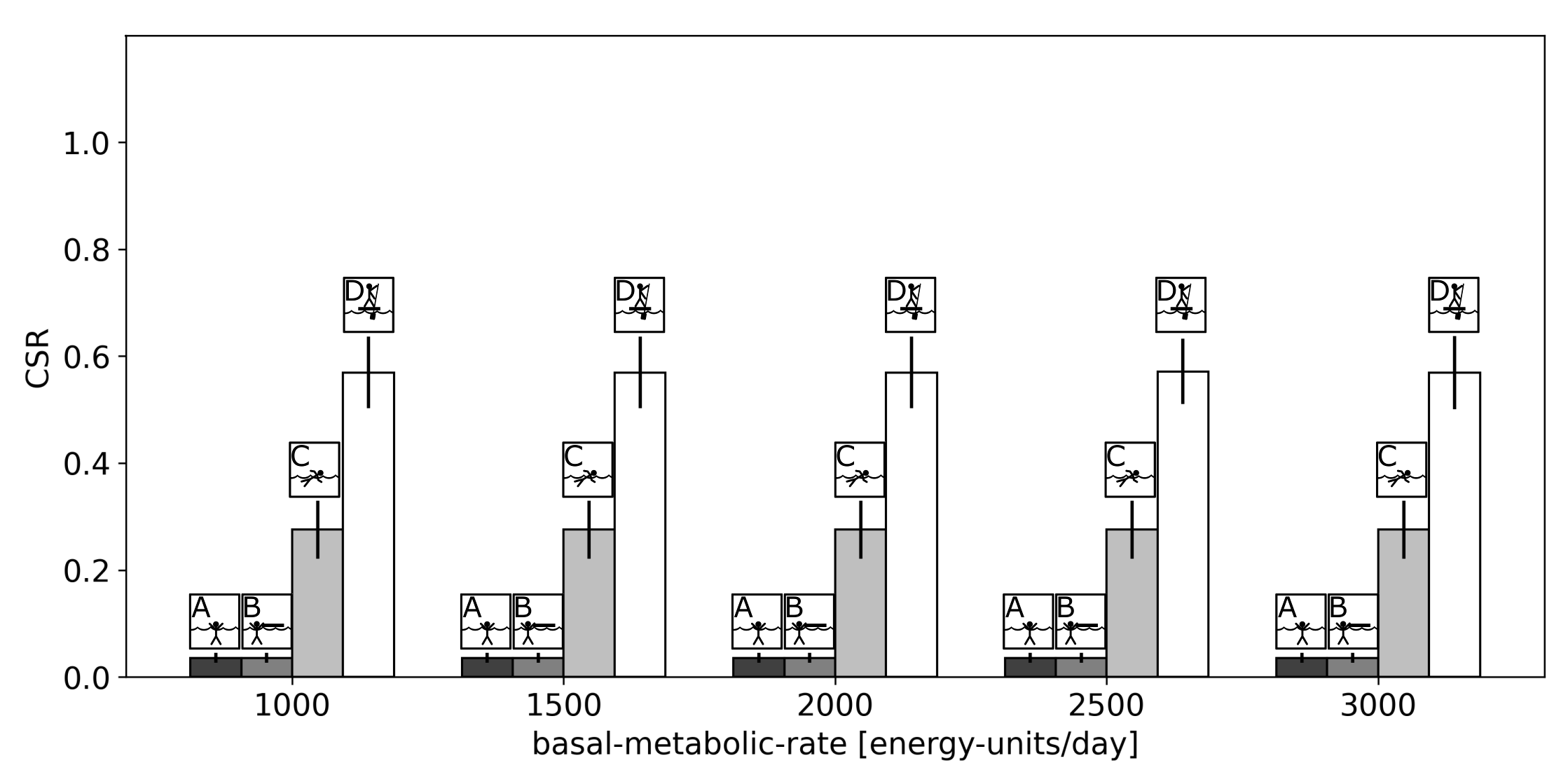

Supplement: S5 Fig — The bars show the average CSRs from 27 simulation runs for each factor level. (TIF) [file pone.0252885.s005.tif]

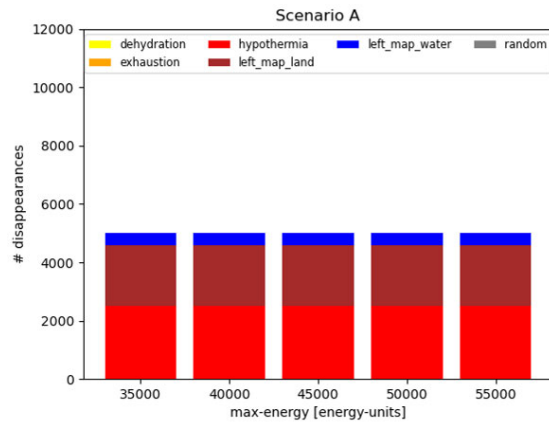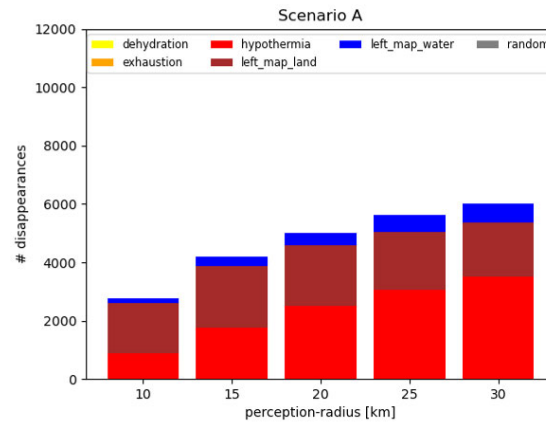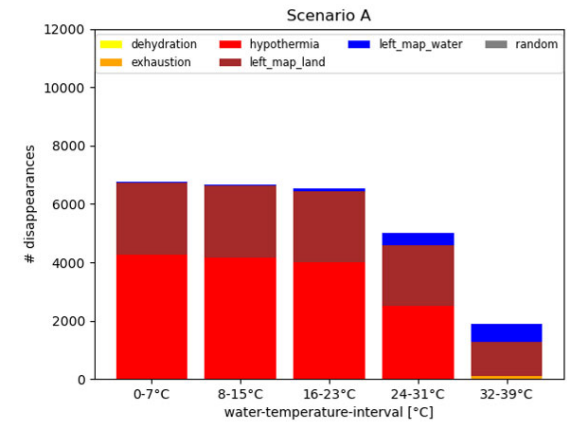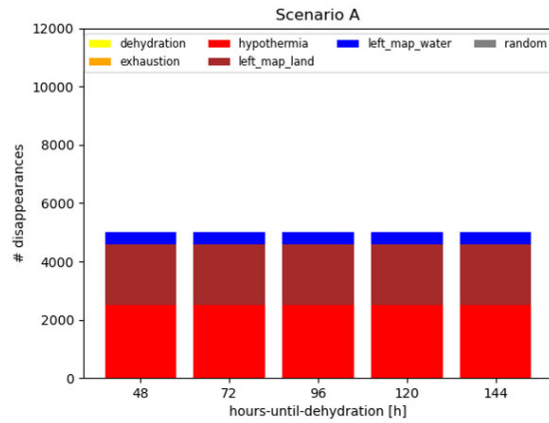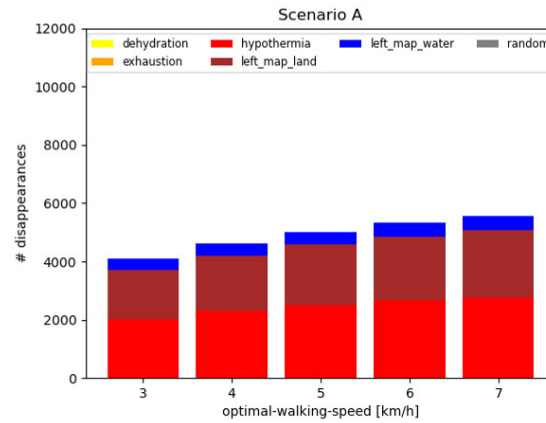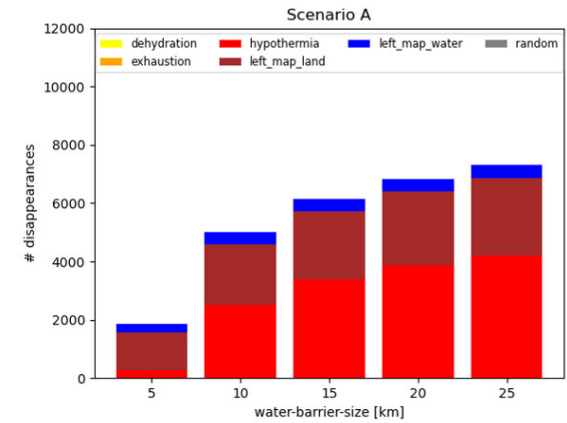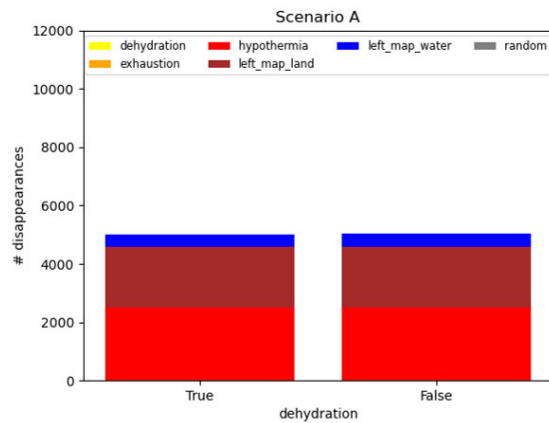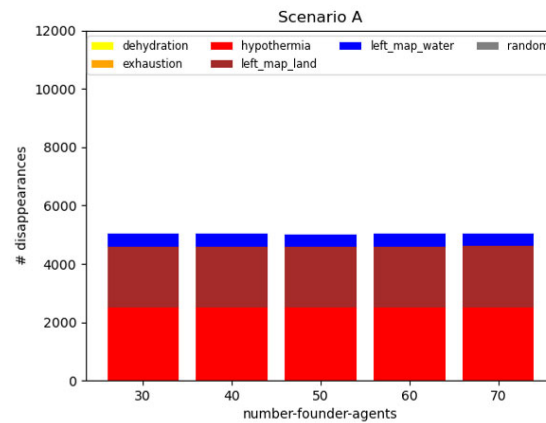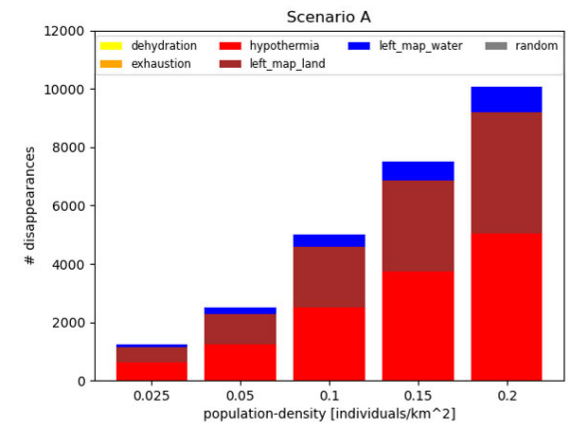

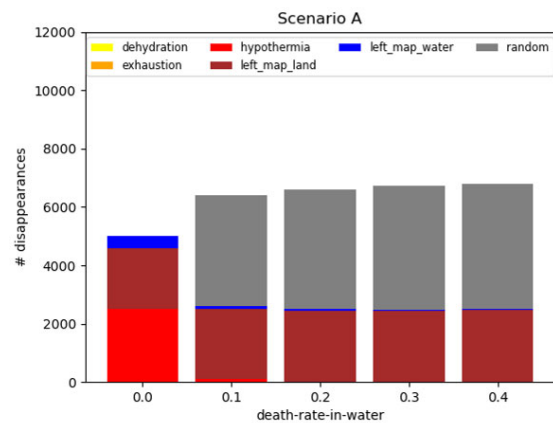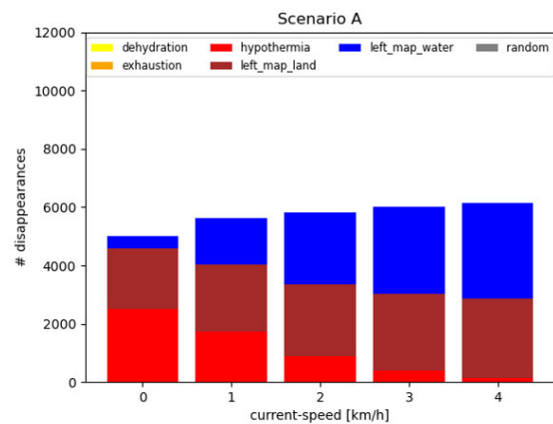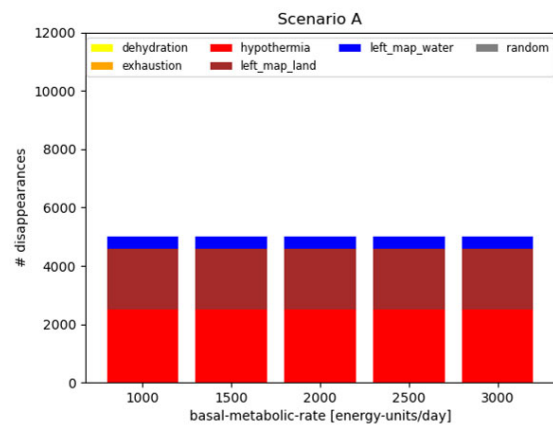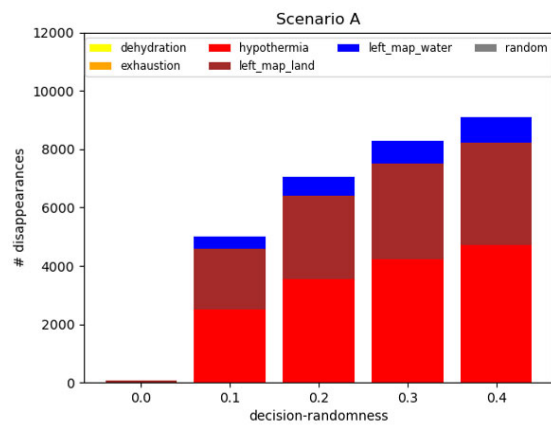

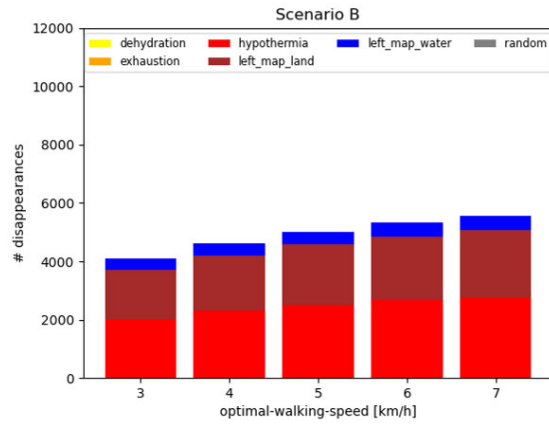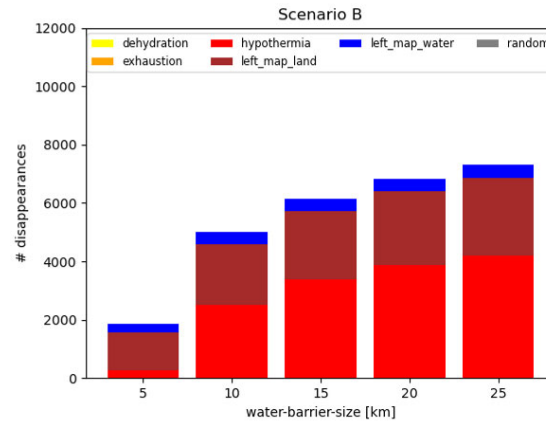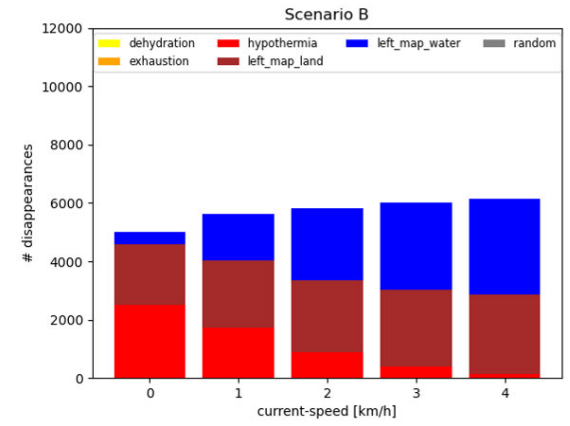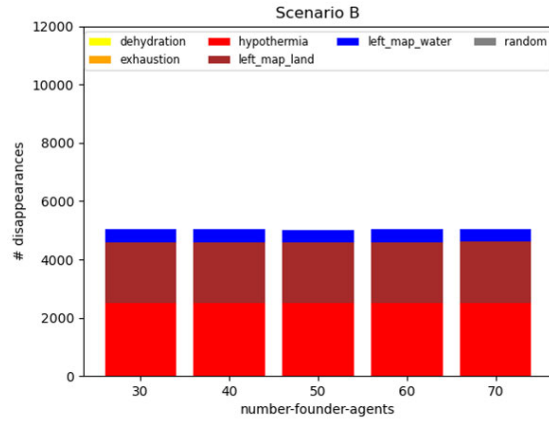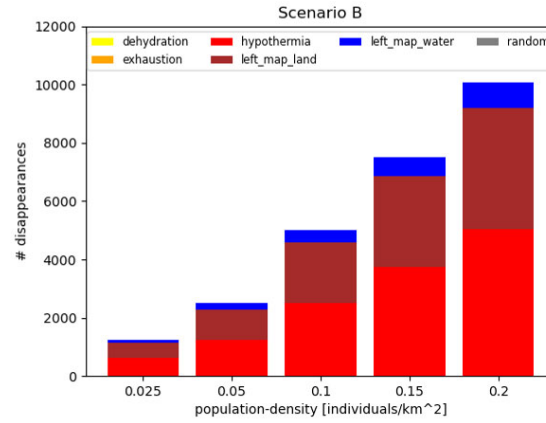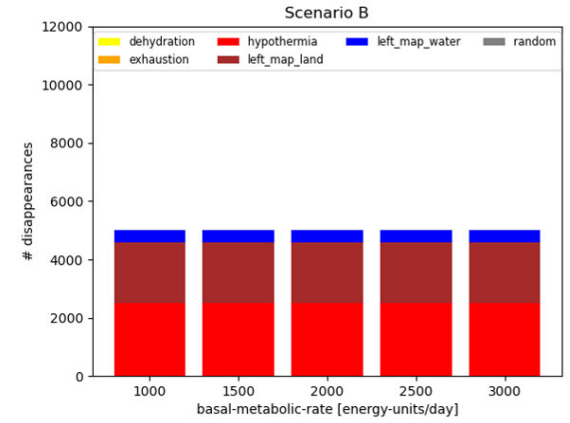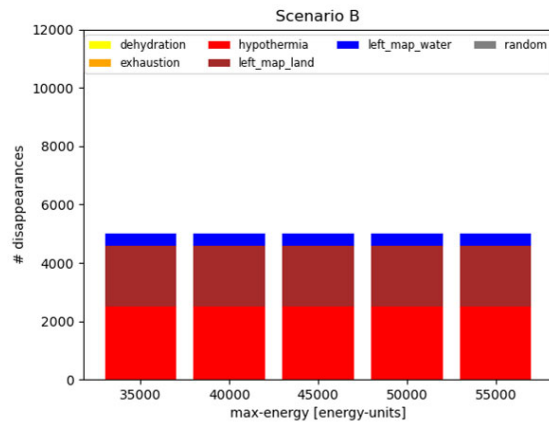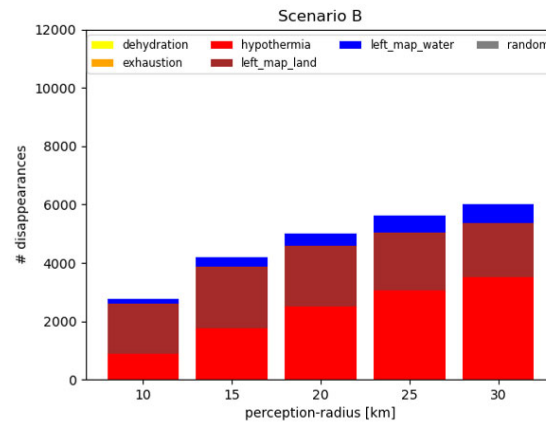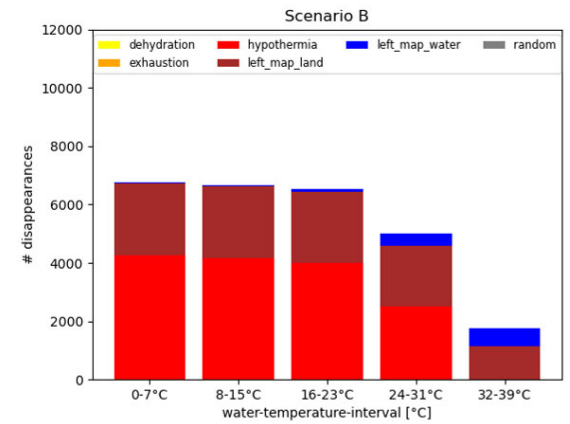

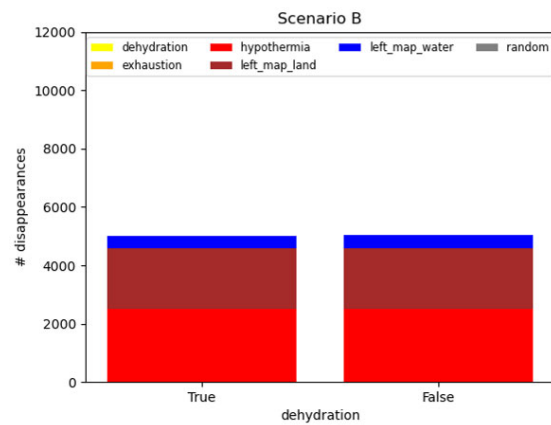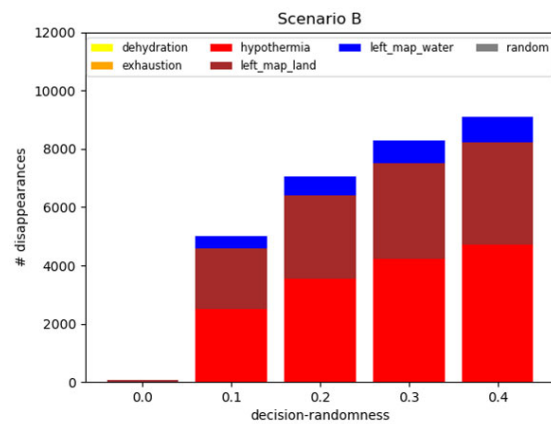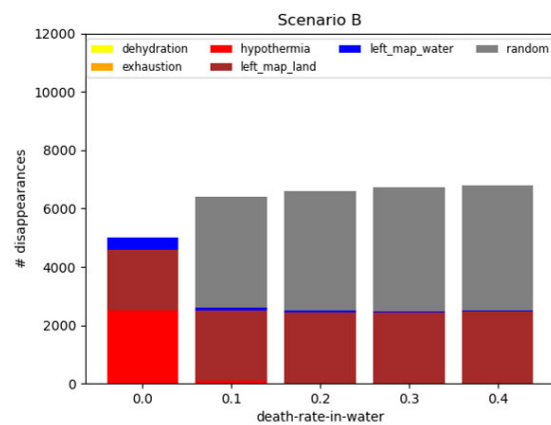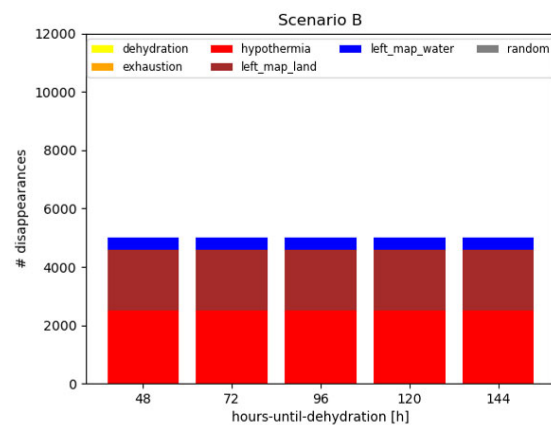

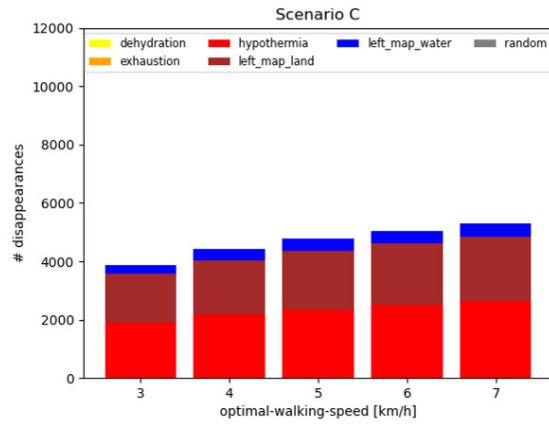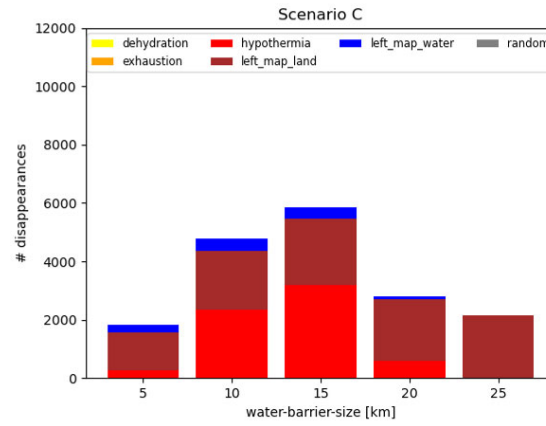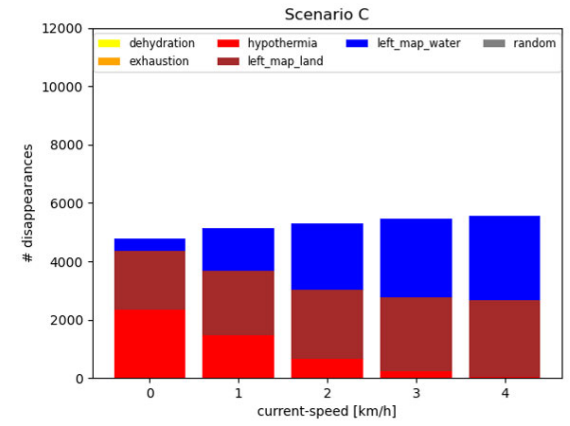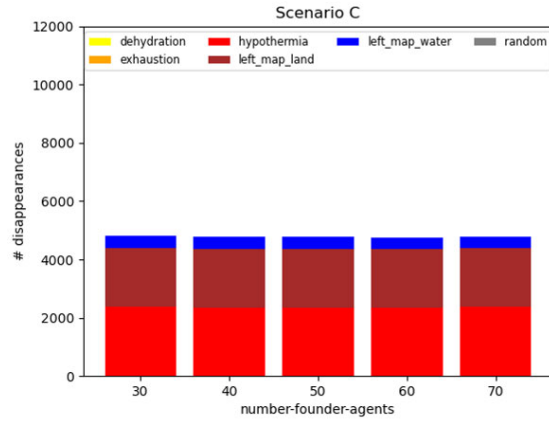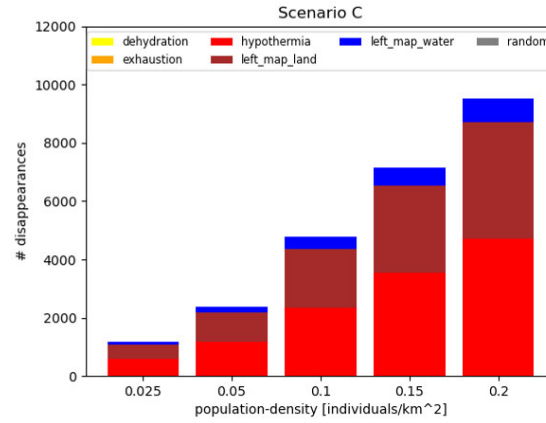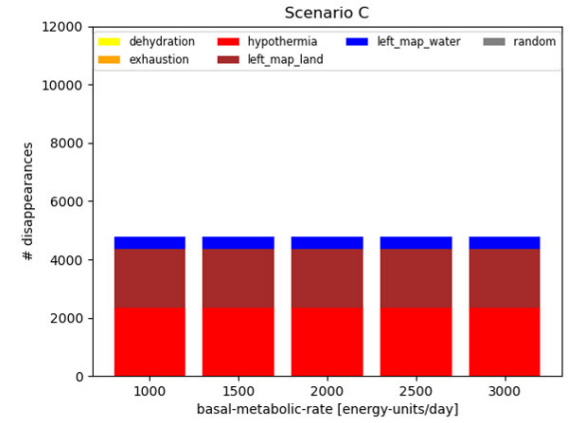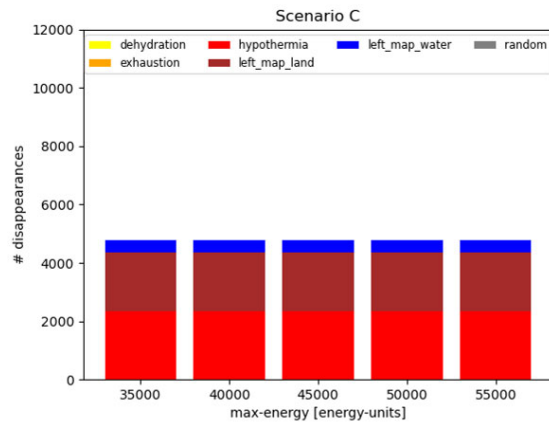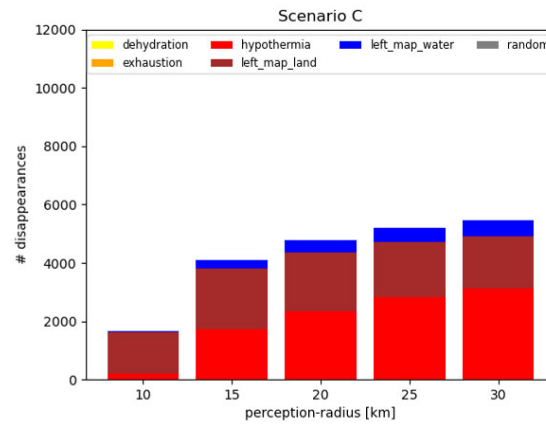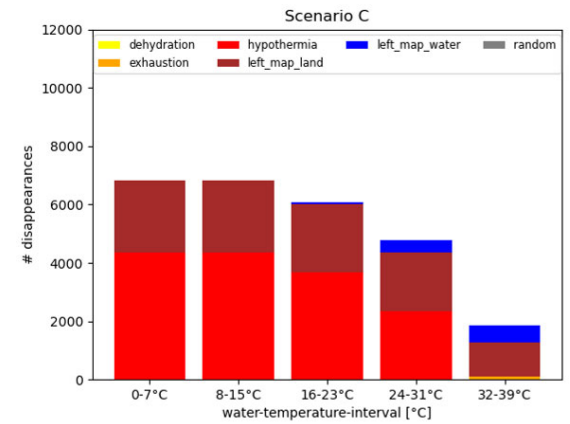

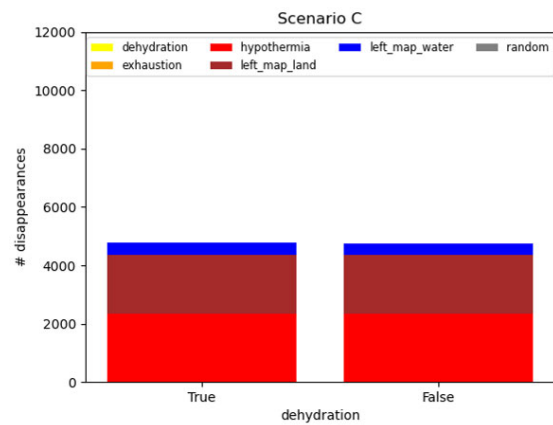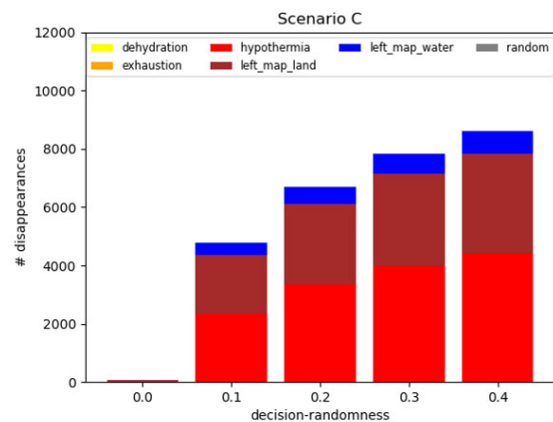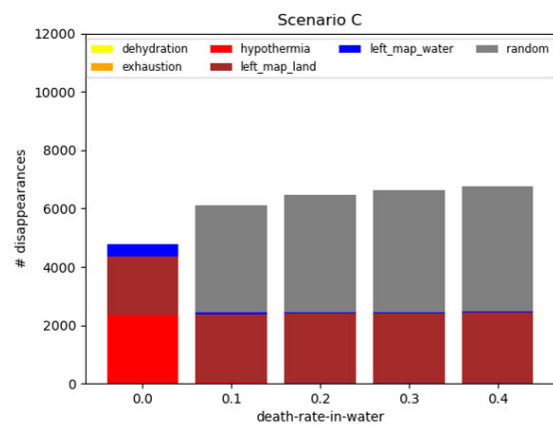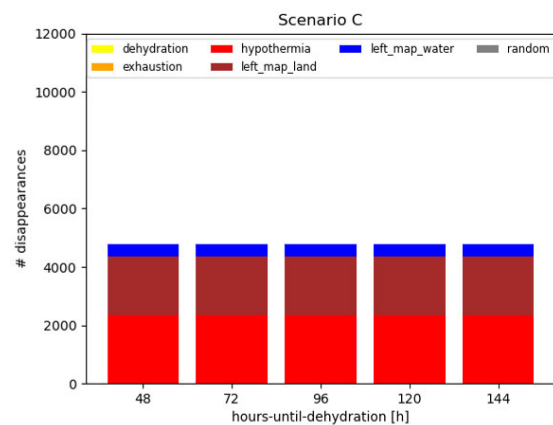

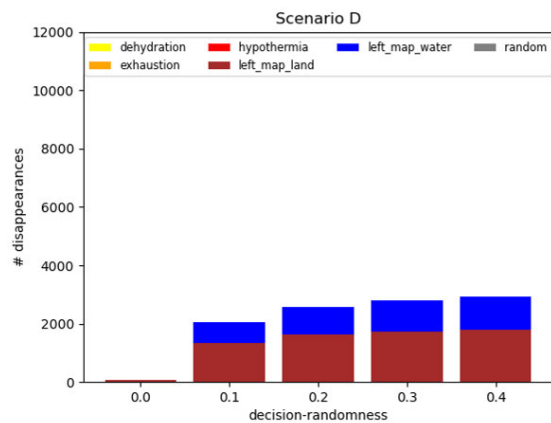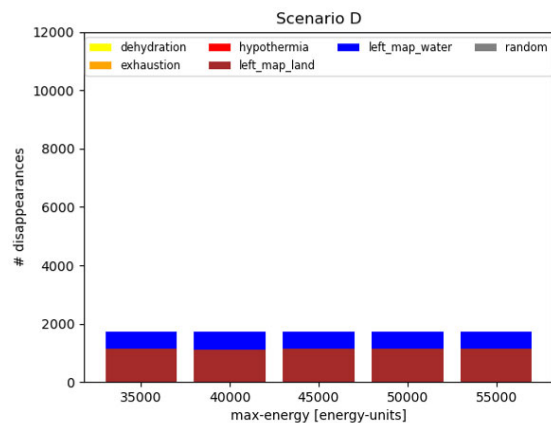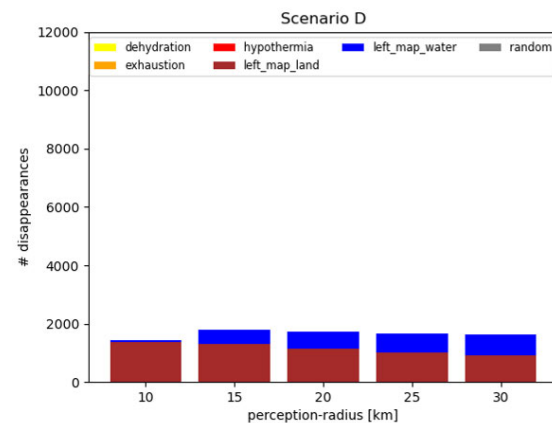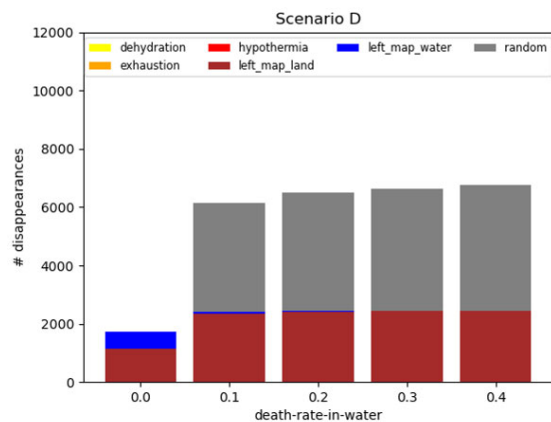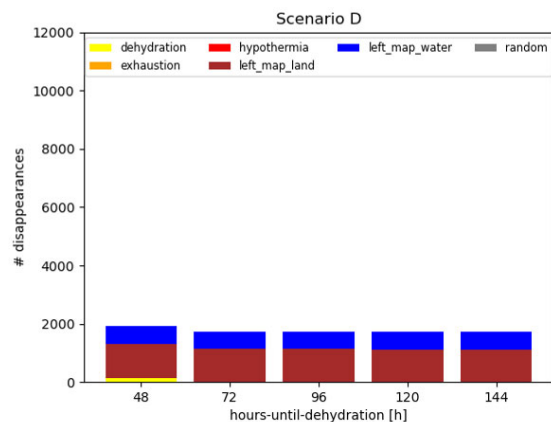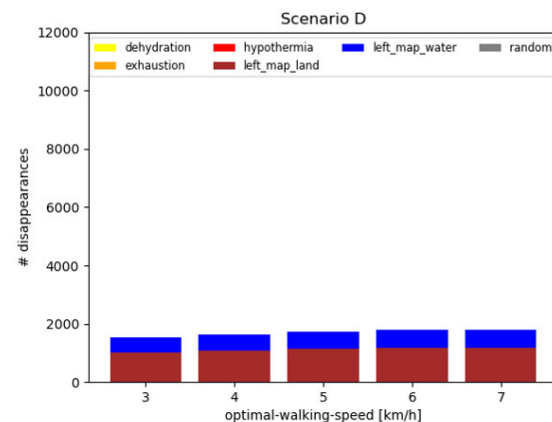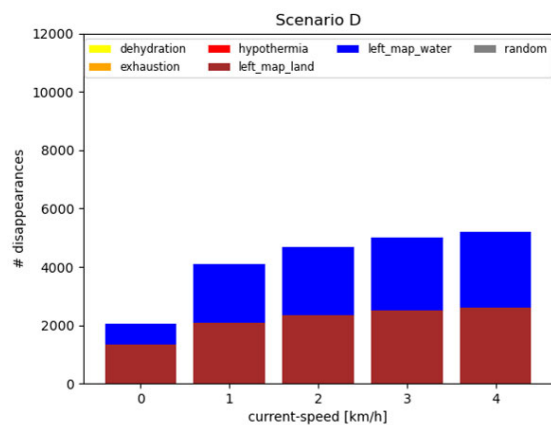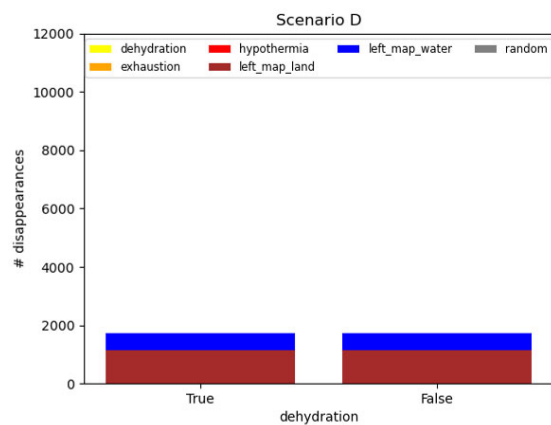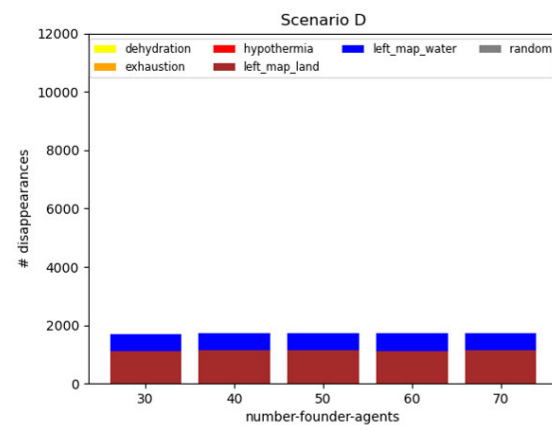

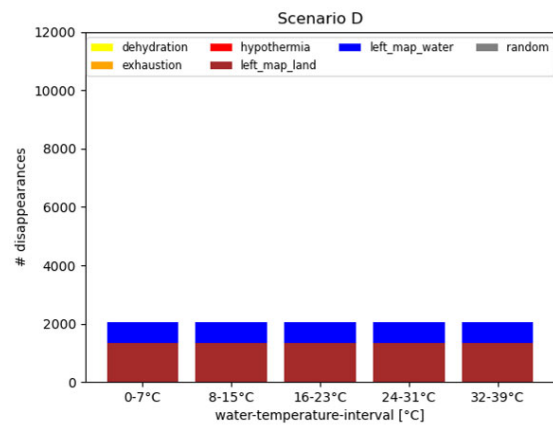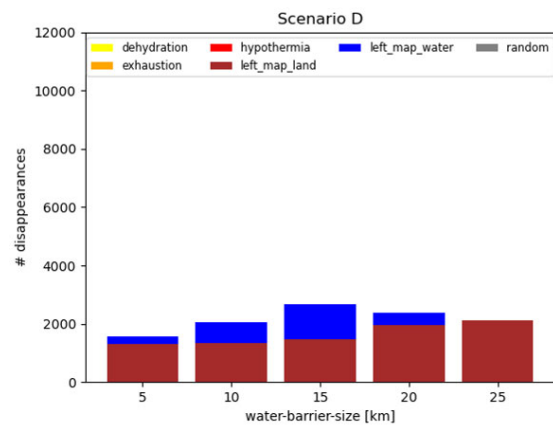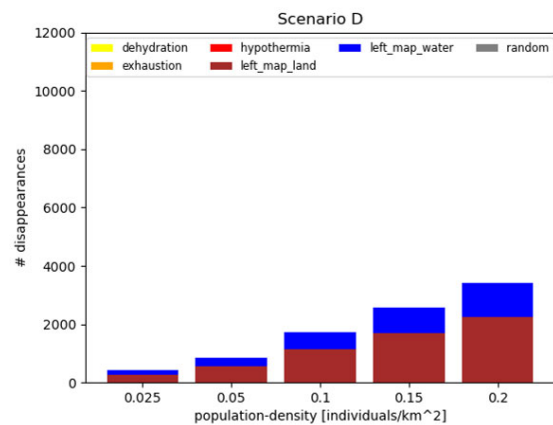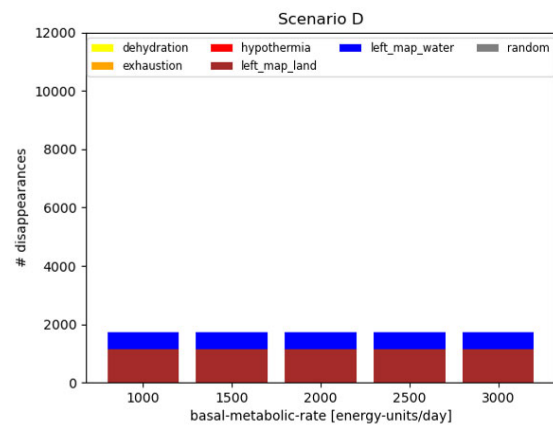

Supplement: S1 File — The bars show the average number of deaths from 27 simulation runs for each factor level. Agents may disappear by leaving the map or when dying. (PDF) [file pone.0252885.s006.pdf]

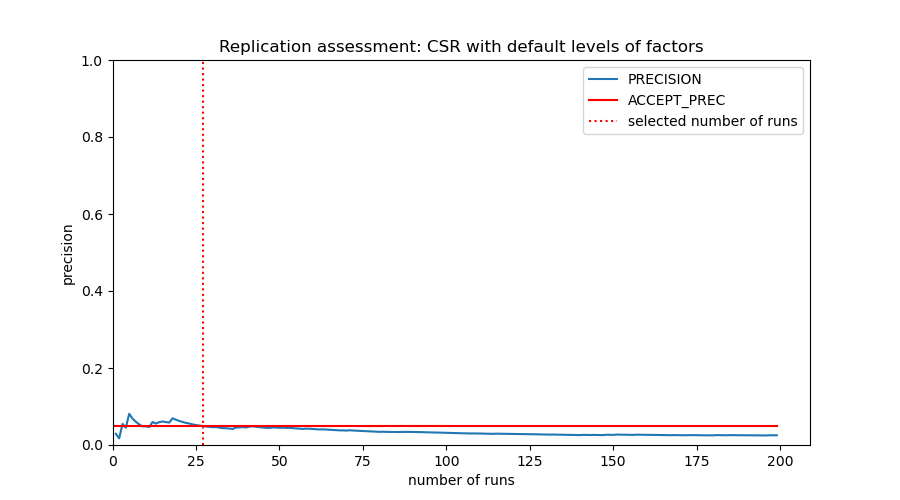

Supplement: S2 File — CSV output of the simulation experiments presented in this paper and Python scripts used for creating the graphs. (ZIP) [file pone.0252885.s007.zip › 01ReplicationAssessment/2ReplAss/ReplicationAssessment.png]
